# Novel cycloneolignans from *Vernicia fordii* with inhibitory effects on over-activation of BV2 cells *in vitro*

**DOI:** 10.1038/s41598-017-14062-z

**Published:** 2017-10-19

**Authors:** Wei-Hong Zhao, Ning Li, Yang Chu, Tao Sun, Jian Wang, Wen-Li Wang, Jia-Yuan Li, Bin Lin, Ru Chen, Yue Hou

**Affiliations:** 1 School of Traditional Chinese Materia Medica, Shenyang Pharmaceutical University; Key Laboratory of Structure-Based Drug Design and Discovery, Ministry of Education, 103 Wenhua Road, Shenyang, 110016 China; 2 Department of Pharmacy, the First Affiliated Hospital of China Medical University, 155 Nanjing north Street, Shenyang, 110001 China; 3 School of Pharmaceutical Engineering, Shenyang Pharmaceutical University; Key Laboratory of Structure-Based Drug Design and Discovery, Ministry of Education, 103 Wenhua Road, Shenyang, 110016 China; 4 College of Life and Health Sciences, Northeastern University, Shenyang, 110819 P.R. China

## Abstract

Novel natural products 7*R*, 8*R*, 7′*R*, 9′*S*-verniciasin A (**1a**), 7*S*, 8*S*, 7′*S*, 9′*R*- verniciasin A (**1b**), 7*R*, 8*R*, 7′*R*, 9′*S*−7′-methoxylverniciasin A (**2a**) and 7*S*, 8*S*, 7′*S*, 9′*R*−7′-methoxylverniciasin A (**2b**) were characterized from the seed capsule of *Vernicia fordii*. And the unique 9-O-9′−7, 9′-cyclo-8, 1′-neolignan skeleton with a seven-membered ring, was identified by extensive spectroscopic analysis. Further the possible biosynthetic pathway was briefly discussed. Interestingly, **1a**, **2a**, **1b** and **2b** all exhibited significant stereoselective inhibitory effects on NO production in LPS-induced BV2 microglia cell. Then the primary mechanism of the bioactivities and stereoselectivity was explored by means of bioassay and molecular docking.

## Introduction

Cycloneolignans, a small group in lignans, mainly exist in the genus *Aniba* (Lauraceae), *Ocatea* (Lauraceae), *Magnolia* (Magnoliaceae) and *Piper* (Piperaceae)^1^. Cycloneolignan is made up of two C6 - C3 units. The reason why it is called cycloneoligan is mainly because the two C6-C3 moieties are connected with a ring, which is commonly formed by a C3 part with another benze ring or two benze rings. Recently, cycloneolignans have attracted more attention because of their structural diversity and significant anti-inflammatory, anti-tumor effects^2–8^. Thus the unique skeleton and impressive anti-inflammatory activities caught our attention^9–12^.

Neuroinflammation induced by sustained overactivation of microglia cells play critical role in the development of Alzheimer’s disease (AD). Excessive activation of microglia cell has been widely acknowledged to be a therapeutic target of AD^13–19^, and inhibition of neuroinflammation is becoming an effective therapeutic measure. However, the most commonly used clinical drugs against neuroinflammation are nonsteroidal anti-inflammatory drugs (NSAIDs) and estrogen, which both bring severe side effects for patients with long-term medication^20^. Therefore, it is more advantageous to pursue natural products with anti-neuroinflammatory effects with low side effects as potential therapeutic agents of AD.

In recent years, the murine microglial cell line was usually selected as *in vitro* model for preliminary biological activity assessment to obtain target compounds. Although the LPS-induce BV2 cell model was widely used for the screening of natural products because of the advantages of large scale and low consumption of natural products. It is really a first step to reveal the toxicity and activity *in vitro*. Especially, it is really important to evaluate the bioavailability and ability to pass the blood-brain barrier (BBB) of the bioactive products or their effective metabolites, because of microglial cells are the resident macrophage-like immune cells in the brain. Therefore *in vivo* evaluation have to be carried out to prove the anti-neuroinflammatory effects.

Employed the preliminary screening method above, we found that natural cycloneolignans from herbal could be potential candidates^21^. In this study, we attempt to find out more novel bioactive products targeted on over-activation of BV2 cells *in vitro*. Then, *Vernicia fordii*, was screened out as a candidate herbal with significant anti-inflammatory effects and abundant lignans. Up to now, there has been few report on *V. fordill*. The previous research^22^ revealed that the extracts of leaves, roots and seeds of plants from *Vernicia* genus, all exhibited anti-inflammatory activities. And “Tong oil”, extracted from the seeds of *V. fordii*, was traditionally used for treatment of burn, scald and wound^23,24^. Moreover, triterpenes, isolated from the leaves of *V. fordii*, presents moderate cytotoxicities in HepG2, A-549 human cancer cells^25^. Meanwhile, the extract of the leaves of *V. fordii* also exhibits antifungal bioactivity^26^. Therefore, bioactivity-guided isolation was carried out based on the chemical profile and bioassay of *V. fordii*. As a result, four novel cycloneolignans were screened out from the husk of *V. fordii*. Herein, we presented the process of isolation, structural elucidation, plausible biosynthesis pathway, stereoselective inhibitory activities on overactivated BV2 cells and primary mechanism of the cycloneolignans **1a**, **1b**, **2a** and **2b**.

## Results

### Structure determination

Verniciasin A (**1**) was obtained as yellow oil (MeOH). Its chemical formula was determined as C_18_H_18_O_7_ by the quasi-molecular ion peak m/z 369.0957 ([M + Na]^+^, calcd. 369.0945 for C_18_H_18_O_7_Na) in HR ESIMS spectrum, which indicated ten degrees of unsaturation. The^1^H-NMR spectrum displayed 18 proton signals, assigning for two aromatic rings, two methylenes, together with three methines. Meanwhile an ABX spin system, observed at *δ*_H_ 6.26, (1 H, dd, J = 8.0, 1.0 Hz), 6.45 (1 H, d, J = 8.0 Hz) and *δ*_H_ 6.54 (1 H, br. s), suggested the existence of a tri-substituted benzene ring. And a tetra-substituted benzene ring was elucidated on the basis of the signals at *δ*_H_ 6.55 (1 H, s) and *δ*_H_ 6.67 (1 H, s). Then 18 carbons were shown in the^13^C-NMR spectrum of **1**, including 12 aromatic carbons, one quaternary carbon (*δ*_C_ 85.8), two methylenes (*δ*_C_ 60.7, 74.9) and three methines (*δ*_C_ 46.9, 71.1, 44.4), which were further confirmed by the HSQC spectrum. Therefore, compound **1** could be elucidated to be a monolignan.

**Figure 1:**
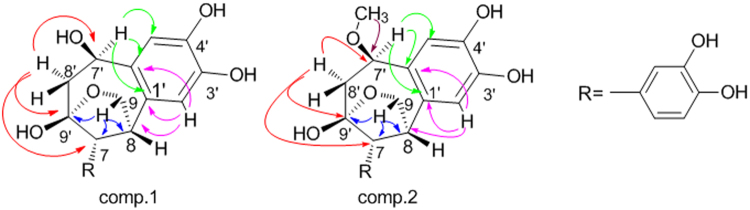
The key HMBC correlations () of compounds **1** and **2**.

The connection of the above fragments were determined by means of extensive analysis of 2D NMR spectra, as shown in Fig. 1. The key HMBC correlations from H-8′a (*δ*_H_ 3.43) to C7′ (*δ*_C_ 71.1), C9′ (*δ*_C_ 85.8), as well as C7 (*δ*_C_ 46.9), indicated that C7 was connected to C9′ directly. The existence of oxygen bridge (C9-O-C9′) was proved by the cross peaks from H-9b (*δ*_H_ 3.49) to C7 (*δ*_C_ 46.9), C8 (*δ*_C_ 44.4) and C9′ (*δ*_C_ 85.8) in the HMBC spectrum. Additionally, the correlations between H-8 (*δ*_H_ 3.25) and C1′ (*δ*_C_ 131.3), C6′ (*δ*_C_ 129.2), combined with the correlations from H-2′ (*δ*_H_ 6.56) to C8, C1′ and C6′ revealed that C8 was attached to C1′ exactly. Thus, it can be inferred that two C6-C3 units were connected by C7-C9′, C8-C1′ and C9-O-C9′ to construct a unique seven-member ring and a furan ring in compound **1**.

**Figure 2:**
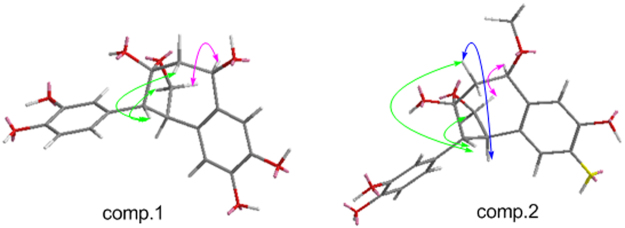
The key NOESY correlations () of compounds **1** and **2**.

**Figure 3:**
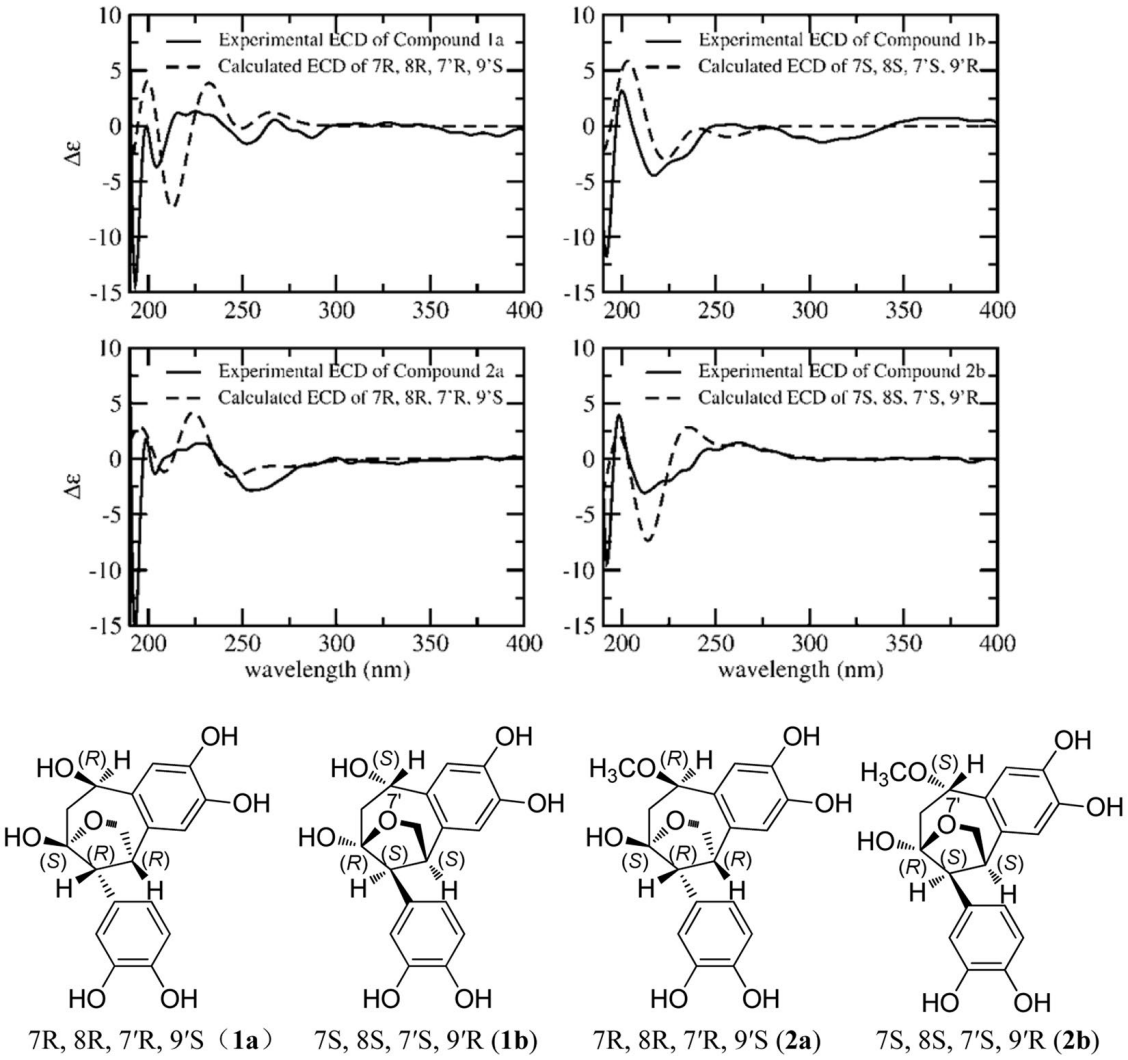
Experimental and calculated ECD spectra of compounds **1a**, **1b**, **2a** and **2b**.

**Figure 4:**
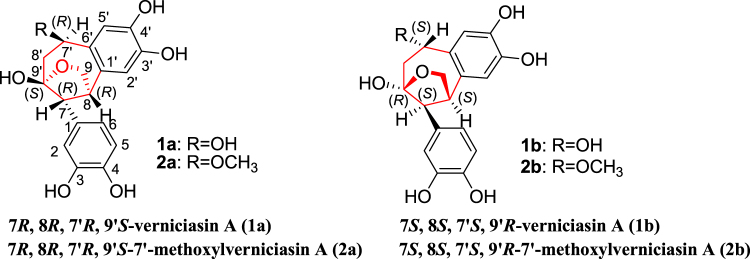
The structures of compounds **1a**, **1b**, **2a** and **2b**.

Then the relative configuration of **1** was further elucidated by the NOE correlations shown in the NOESY spectrum (Fig. 2) and coupling constant of H-7, observed as J = 2.9 Hz in^1^H-NMR spectrum, which indicated the same orientation of H-7 and H-8. Furthermore, the NOE correlations originated from *δ* 3.90 (H-7′) to *δ* 3.97 (H-9*α*), from *δ* 3.63 (H-7) to 3.99 (H-8′*β*) and 3.49 (H-9*β*) revealed the relative configuration of **1** as shown in Fig. 2. Due to the difficulty in recrystalization of natural neolignans, no suitable crystals of **1** and **2** were yielded despite many attempts. We therefore employed ECD calculation to determine their absolute configurations (Fig. 3)^27,28^. The ECD spectrum of **1a** exhibited a negative cotton effect at 240–270 nm and a positive cotton effect at 220–240 nm. While the ECD spectrum of **1b** displayed exactly the reverse cotton effects compared with those of **1a**. Finally, the absolute configurations of C7, C8, C7′ and C9′ in **1a** and **1b** were determined to be 7 *R*, 8 *R*, 7′*R*, 9′*S* and 7 *S*, 8 *S*, 7′*S*, 9′*R* respectively as shown in Fig. 4.

Compound **2** was purified as yellow oil (MeOH). The chemical formula of **2** was indicated to be C_19_H_20_O_7_ on the basis of *m/z* 383.1106, [M + Na]^+^ (calcd. 383.1101 for C_19_H_20_O_7_Na) displayed by HR-ESI-MS, which required 10 indices of hydrogen deficiency. A side-by-side comparison of NMR data of **2** with those of **1** (see Table 1) revealed that both **2** and **1** have the same 9-O-9′−7, 9′-cyclo-8, 1′-neolignane skeleton. However, the ^1^H-NMR spectrum indicated an additional methoxy group (*δ*_OCH3_ 3.30, 3 H, s) in compound **2** instead of the hydroxyl group substituted at C7′ in compound **1**. The HMBC long-range coupling (Fig. 1) between OCH_3_–7′ and C7′ proved the methylation of hydroxyl group at C7′. Further, the planar structure was demonstrated by HSQC and HMBC spectra. Similarly, compound **2** was also purified as a pair of enantiomers **2a** and **2b** by chiral separation.

**Table 1:** The NMR data of compounds 1 and 2 measured in DMSO-*d*_6_.

No.	Comp. 1		Comp. 2	
	δ H	δ C	δ H	δ C
1	—	128.5	—	128.3
2	6.54 (br. s)	116.4	6.54 (br. s)	116.3
3	—	144.5	—	144.8
4	—	143.4	—	143.9
5	6.45 (d, 8.0)	114.9	6.46 (d, 8.1)	114.6
6	6.26 (dd, 8.0, 1.0)	119.1	6.27 (br. d, 8.1)	119
7	3.63 (d, 2.9)	46.9	3.65 (d, 2.7)	47.5
8	3.25 (m)	44.4	3.26 (m)	44.3
9	3.49 (br. d, 6.2);	74.9	3.49 (br.d, 6.8);	75.1
	3.96 (m)		3.96 (m)	
1′	—	131.3	—	132.3
2′	6.55 (s)	113.1	6.57 (s)	113.6
3′	—	144.4	—	144.6
4′	—	143.7	—	143.6
5′	6.67 (s)	115.3	6.60 (s)	115
6′	—	129.2	—	128.3
7′	3.90 (br. d, 8.6)	71.1	3.84	80.2
8′	3.43 (br. d, 12.2);	60.7	3.51(m);	60.4
	3.99 (m)		3.95 (m)	
9′	—	85.8	—	86.8
-OCH_3_	—	—	3.30 (s)	57.3

The relative configuration of **2**, was deduced from the NOE correlations observed in the NOESY spectrum as shown in Fig. 2. NOE correlations from H-7′ to H-9a, H-7 to H-9b and H-8′b, together with correlations from H-8 to H-8′b, indicated the same configuration with that of **1**. After that the absolute configurations of **2a** and **2b** were established by calculated ECD spectra (Fig. 3). Therefore, the structures of **2a** was finally identified as 7 *R*, 8 *R*, 7′*R*, 9′*S*−7′-methoxylverniciasin A, and **2b** as 7 *S*, 8 *S*, 7′*S*, 9′*R*−7′-methoxylverniciasin A.

### Extracts of *V. fordii* inhibit production of NO in LPS-induced BV-2 cells

The inhibitory effects and cytotoxicities of the extracts and purified compounds were assayed using Griess and MTT methods respectively. And the results indicated that the 70% EtOH extract of seed capsule from *V. fordii* could significantly inhibit the production of NO in LPS-induced BV2 cells (IC_50_ 7.03 ± 0.99 μg/ml) with cytotoxicity at 100 μg/ml (IC_50_79.63 ± 1.23 μg/ml). To reveal the effective fraction, PE (petroleum ether), EtOAc (ethyl acetate) and *n*-BuOH (*n*-butyl alcohol) extracts were further evaluated, and the results were listed in Table 2. Then, the EtOAc extract was finally determined as effective composition because of its significant inhibitory activity with IC_50_ values at 10.70 ± 1.23 μg/ml (cytotoxicity IC_50_ > 100 μg/ml) and high weight percentage (69.4%) in the total 70% EtOH extract. It is worth noting that all the extracts showed no cytotoxicity at their effective concentrations.

**Table 2:** Inhibitory Effects and cytotoxicities of extracts and identified novel neolignans from the seed capsule of *V. fordii* on NO production by LPS-activated BV2 cells.

Sample	Inhibitory effect	Cytotoxicities
	IC_50_	IC_50_
Ext-1	7.03 ± 0.99 μg/ml	79.63 ± 1.23
Ext-2	22.05 ± 1.58 μg/ml	>100
Ext-3	10.70 ± 1.23 μg/ml	79.54 ± 1.55
Ext-4	44.12 ± 1.61 μg/ml	>100
Compound 1	1.46 ± 0.91 μM	>100
Compound 2	17.35 ± 0.87 μM	>100

Ext-1: 70% ethanol crude extract; Ext-2: petroleum ether extract; Ext-3: ethyl acetate extract; Ext-4: *n*-butanol extract.

### Cycloneolignans inhibit production of NO in LPS-induced BV-2 cells

**Figure 5:**
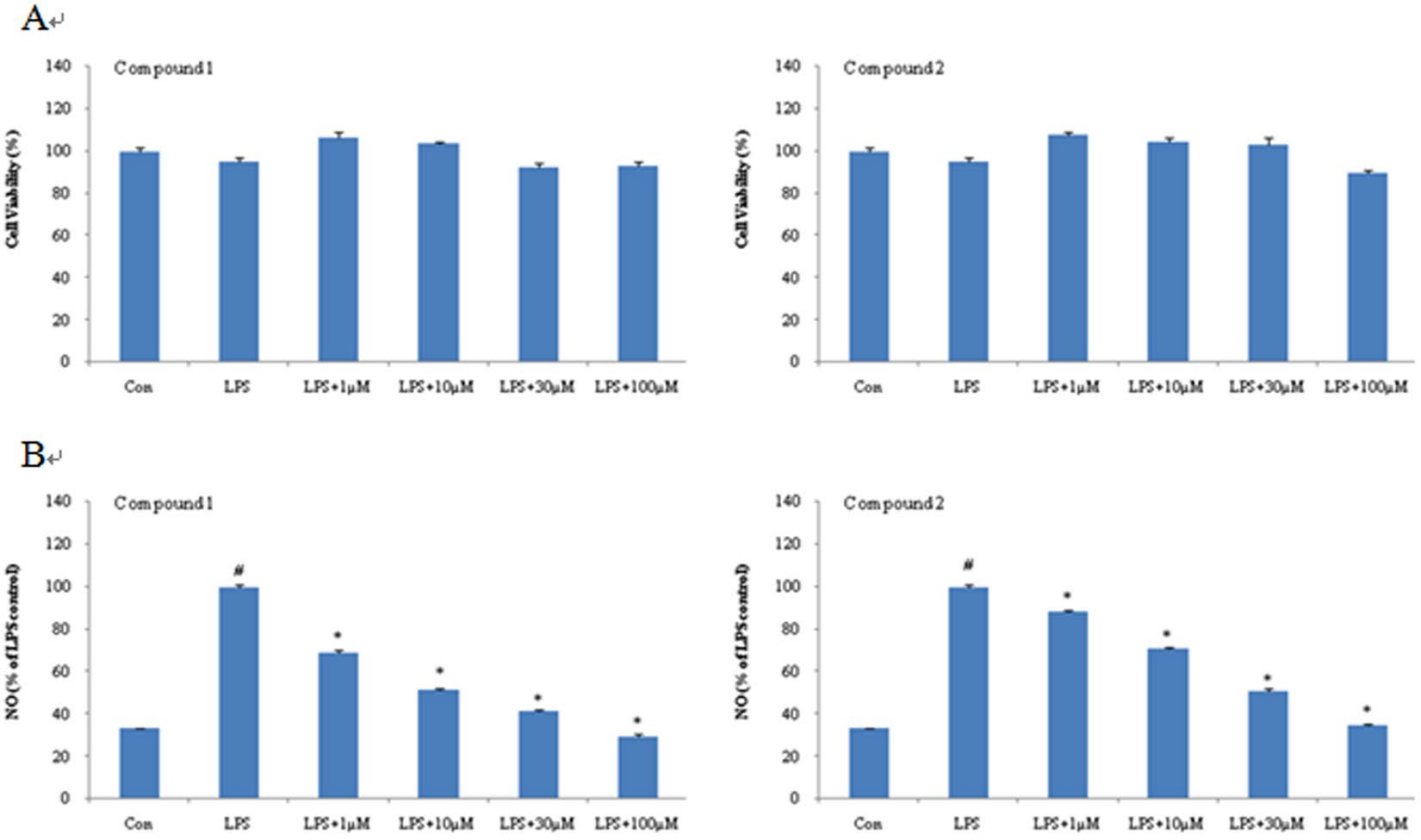
Effect of compounds **1** and **2** on LPS-induced NO production in microglial cells. (**A**) BV-2 cells were treated with compounds **1** and **2** in the presence of LPS (100 ng/ml) for 24 h at the indicated concentrations. Cell viability was detected by MTT assay. (**B**) BV-2 cells were treated with compounds **1** and **2** in the presence of LPS (100 ng/ml) for 24 h. NO production was tested by Griess reaction. Data are expressed as means ± SEM (n = 3). ^#^P < 0.05 compared with the control group, *P < 0.05 compared with LPS group.

As exhibited in Fig. 5 and Table 2, compounds **1** and **2** showed significant dose-dependent inhibitory effects on NO production in LPS-induced BV-2 cells without obvious cytotoxicities at the effective concentrations. Compound **1** was found to show inhibitory effect with the IC_50_ value at 1.46 ± 0.91 μM, while compound **2** was at 17.35 ± 0.87 μM. Meanwhile, compounds **1** and **2** did not exhibit obvious cytotoxicities at the tested concentration from 1 μM to 100 μM (IC_50_ >100 μM).

### Molecular docking of cycloneolignans with iNOS

**Figure 6:**
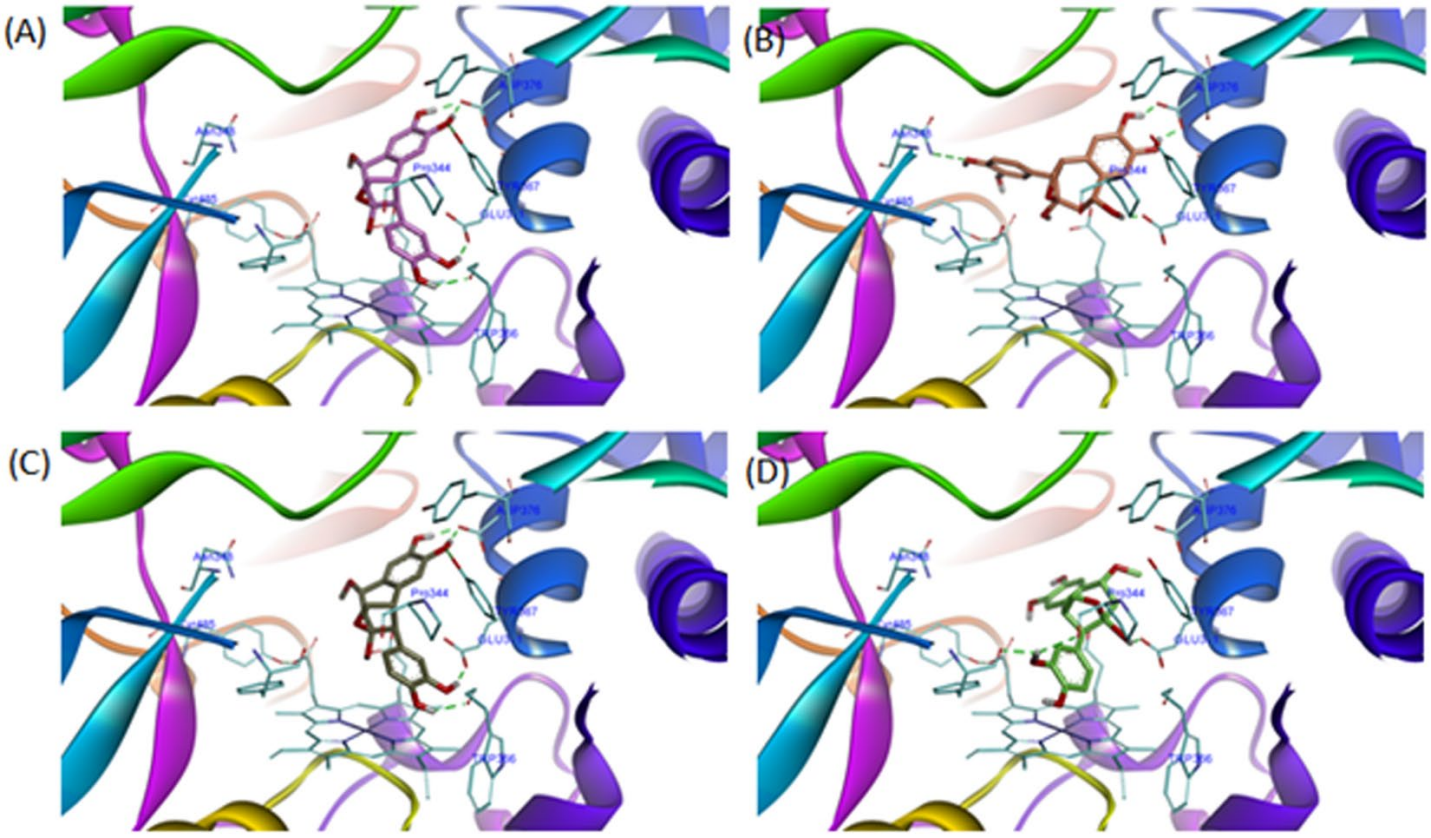
Binding patterns of compounds 1a(A, pink), 1b(B, orange), 2a(C, brown) and 2b(D, green) within the iNOS binding pocket. The protein structure was shown as ribbon, compounds were displayed as sticks, HEME and residues were displayed as thin stick.

In order to clarify the plausible mode of interaction between iNOS (inducible nitric oxide synthase) and the bioactive cycloneolignans (**1a**, **1b**, **2a**, and **2b)**, molecular docking was performed to measure their relative binding energies and localize binding sites with iNOS. And the results were shown in Fig. 6. The calculated binding energies of **1a**, **1b**, **2a** and **2b** were −10.62 kcal/mol, −8.17 kcal/mol, −10.96 kcal/mol and −8.73 kcal/mol, which were all lower than that of co-crystal ligand imidazopyridine (−8.01 kcal/mol). It seems that the spatial orientation of 7 *R*, 8 *R*, 7′*R*, 9′*S* isomers (**1a** and **2a**) interacted with iNOS in better conditions.

### Effect of cycloneolignans on LPS-induced IL-6 and IL-1βexpression in BV-2 cells

**Figure 7:**
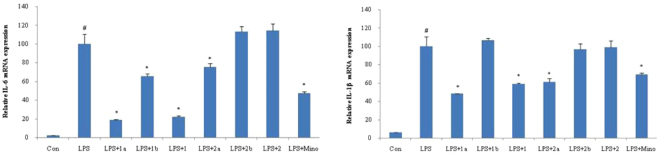
Effects of compounds 1, 1a, 1b, 2, 2a and 2b on LPS-induced IL-6 and IL-1β mRNA expression in microglial cells. BV-2 microglial cells were pretreated with compounds1, 2, 1a, 1b, 2a and 2b (10 μM) for 2 h and then stimulated with LPS (100 ng/mL). Total RNA was isolated 4 h after LPS treatment, the mRNA levels of IL-6 and IL-1β were measured by qRT-PCR. Data are expressed as means ± SEM (n = 3). ^#^P < 0.05 compared with the control group, *P < 0.05 compared with LPS group. Mino stands for minocycline.

The effects of the racemates (**1** and **2**) and optical pure isomers (**1a**, **1b**, **2a** and **2b**) on LPS-induced mRNA expression of proinflammatory cytokines IL-6 and IL-1*β* in BV2 cells were tested by Real-time PCR. As shown in Fig. 7, we could find that racemic compound **1** suppressed IL-6 and IL-1*β* mRNA expression in over-activated BV2 cells. However, compound **2** did not show inhibitory effect at the tested concentration (10 μM). Although racemic compound **2** was not active, the optical pure compound **2a**, purified from **2**, exhibited moderate inhibitory effect on both IL-6 and IL-1*β* mRNA level. However optical pure **1a** presented significant inhibitory action on IL-6 and IL-1*β* mRNA expression at 10 μM, while **1b** did not display inhibitory activity. Therefore, the SAR and stereo-selectivity of cycloneolignans on inhibitory effects against overactivation of BV2cells *in vitro* was suggested as followed.

### The SAR and stereo-selectivity of cycloneolignans on inhibitory effects against overactivation of BV2cells *in vitro*

According to the data shown in Fig. 7, racemate **1** expressed stronger inhibitory effect than that of **2**, which indicated that 7′-hydroxyl group could improve the anti-inflammatory effect. Also, the difference between the inhibitory effects of optical pure isomers (**1a**, **1b**, **2a** and **2b**) revealed the obvious stereo-selective bioactivities of the novel skeleton. Obviously, 7 *R*, 8 *R*, 7′*R*, 9′*S* configuration (**1a** and **2a**) exhibited much stronger inhibitory effects than those of 7 *S*, 8 *S*, 7′*S*, 9′*R* isomers (**1b** and **2b**). The possible reason for the stereo-selectivity could also be concluded from the above mentioned molecular docking results between the different isomers and iNOS.

### Plausible biosynthetic pathway of cycloneolignans

**Figure 8:**
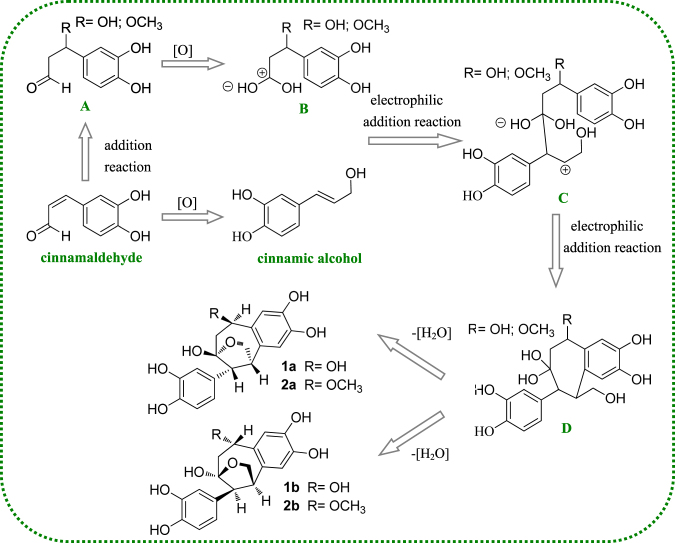
Plausible biosynthetic pathway of **1a**, **1b**, **2a** and **2b**.

Based on characteristics of the identified structures, a plausible biosynthetic pathway was proposed as shown in Fig. 8. The biosynthetic precursor of the novel skeleton could be cinnamic aldehyde. The formation of key intermediates were proposed to be from two steps of electrophilic additions, coupled with the other C6-C3 units. Finally, a dehydration reaction occurred between C9′-OH and C9-OH to afford compound **1**. After that, a methylation at C-7′OH could eventually convert compound **1** to **2**.

## Discussion

Lignans, as an important type of natural products, exist widely in herbal plants. They have attracted much attention of scientists because of their diverse bioactivities and skeletons. While, the novel cycloneolignans are a small group in natural lignan family. Cycloneolignan skeleton is consisted of two C6-C3 units, connected through a ring. Up to now, a limited number of cycloneolignans have been found from some medical plants of Lauraceae, Piperaceae and Magnoliaceae family^6,8,29,30^. And the reported natural cycloneolignans exhibited significant anti-inflammatory and anti-tumor effects^6,8,29,30^. Therefore the novelty of cycloneolignan framework and significant anti-inflammatory activities stimulate our interest to further investigate more bioactive cycloneolignans with unique structures. Then, *V. fordii*, screened out as a candidate with inhibitory effect on over-activation of BV2 cells in our previous work, was subjected to phytochemical research and bioassay successively. As a result, (±) verniciasin A (**1**) and (±) methoxylverniciasin A (**2**), with a distinct 9, 9′-epoxy-7, 9′-cyclo-8, 1′-neolignan skeleton were identified on the basis of extensive spectral analysis. Interestingly, the two C6-C3 units were assembled by two rings, including a tetrahydrofuran deduced by C9-O-C9′ bridge and a seven - membered carbocyclic ring formed by connection between C7 and C 9′, C8 and C1′ respectively. However, it is the first time to find natural 9, 9′-epoxy-7, 9′-cyclo-8, 1′-neolignan. The novel skeleton can enrich cycloneolignan group in lignan family.

Microglia cells, as the resident immunocytes in central nervous system, play a central role in defending the brain against injury or diseases^31^. Under physiological conditions, microglia can support and nourish nervus centralis. Once, the external stimuli, such as LPS, Aβ *et al*., continuously attack, microglia would be activated and then release pro-inflammatory molecules including NO, IL-1β, IL-6 and so on. In turn the pro-inflammatory cytokines will further stimulate the microglia cells. Then the vicious circle can cause neuronal damage, pathological changes and neurodegenerative diseases^32–34^. Therefore, the over-activation of microglial is a very promising therapeutic target for AD^33^. However, non-steroidal anti-inflammtory agents and estrogenic drugs are still the most commonly used clinical drugs in resisting neuroinflammation and the serious side effects severely limited their long-term administration and clinical therapeutic effects^20^. Thus, pursuing natural neuroinflammatory inhibitors with low side effects has become a necessity. Fortunately, cycloneoligans could become candidates as potential therapeutic agents for neurodegenerative diseases targeted on over-activated microglial^21^.

NO is mediated in mammals by the calcium-calmodulin controlled isoenzymes eNOS (endothelial NOS) and nNOS (neuronal NOS). The inducible isoform, iNOS, is involved in immune response and production of NO as an immune defense mechanism. Microglia express an inducible isoform of NOS (iNOS)^35,36^. To investigate the *in vitro* anti-inflammatory effects of the extracts of *V. fordii*., we monitored NO production in LPS-induced BV-2 microglial cells. The total 70% EtOH extract of *V. fordii* significantly inhibited production of NO with IC_50_ values at 7.03 ± 0.99 μg/ml. Further study revealed that the EtOAc extract significantly reduced LPS-induced NO production with IC_50_ values at 10.70 ± 1.23 μg/ml, while the PE extract and *n*-BuOH extract showed moderate inhibitory activities with IC_50_ values at 22.05 ± 1.58 μg/ml and 44.12 ± 1.61 μg/ml, respectively. Combined the bioassay results with weight percentage of the extracts, it was suggested that the EtOAc extract (69.4% in the total 70% EtOH extract of *V. fordii*.) contained the major active ingredients which are responsible for the inhibitory effects exhibited by the herbal. Furthermore, the novel cycloneolignans (**1a**, **1b**, **2a** and **2b**) were purified from the effective extract and their inhibitory action on over-activated BV2 cells were evaluated successively. The two racemates (**1** and **2**) both showed significantly inhibitory effects on LPS-induced NO production in BV2 cells, and **1** (IC_50_: 1.46 ± 0.91 μM) exhibited much stronger inhibitory effect than that of **2** (IC _50_: 17.35 ± 0.87 μM). The possible reason for the stereo-selective bioactivity was investigated by molecular docking between the isomers and iNOS. The calculated binding energy (−10.62 kcal/mol for **1a**, −8.17 kcal/mol for **1b**, −10.96 kcal/mol for **2a**, −8.73 kcal/mol for **2b**) indicated that the spatial orientation of 7 *R*, 8 *R*, 7′*R*, 9′*S* isomer might interact with iNOS in the better condition.

In respect to the reliability of the predicted binding architectures, it’s essential to provide evidences on the reliability of the method in reproducing the binding pose of known ligands. Therefore, we collected several known ligands from literature to build a test set. And those molecules were docked onto iNOS binding pocket to validate the model and check the capability of reliably reproducing the binding mode and binding affinity of ligands (Figure S20–S24 and Table S1).

It could be informative to better understand the mechanisms of inhibition a tentative elucidation on how and why the differences of molecules affect the binding site recognition and the binding affinity as well. Therefore, molecular docking strategy was used to predict the binding patterns and affinities of different isomers. It’s supposed that the more interactions between protein-ligand formed, the more potent the ligand would be. Those interactions include hydrogen bonds (HB), hydrophobic interactions (HP), aromatic interactions (AR), etc. As shown in the docking results, more interactions were formed for both compounds 1a and 2a, with better binding energies of −10.62 kcal/mol and −10.96 kcal/mol, respectively. Nevertheless, less interactions were formed for both compounds 1b and 2b, with worse binding energies of −8.17 kcal/mol, −8.73 kcal/mol, respectively. Therefore, it’s concluded that the number of protein-ligand interactions and the values of binding energies are consistent with activity data. Meanwhile, it’s also shown that residues Trp366, Tyr367, Glu371 and Asp376 are crucial for ligand binding, which formed hydrogen bonds with both 1a and 2a, neither with 1b or 2b (Fig. 6).

Pro-inflammatory cytokines IL-1*β*, IL-6 are also important markers for over-activation of microglia cells^37^. Therefore, the mRNA expression of pro-inflammatory cytokines was determined in LPS-induced BV2 cells, which are treated with the racemates (**1** and **2**) and 4 optical pure isomers (**1a**, **1b**, **2a** and **2b**) respectively. The difference between the anti-inflammation effects of the purified components indicated the SAR and stereo-selectivity of 9, 9′-epoxy-7, 9′-cyclo-8, 1′-neolignan skeleton for their anti-inflammatory action. The fact that racemate **1** showed obvious inhibition on mRNA expression indicated that 7′-OH plays a key role in inhibiting the expression of IL-6 and IL-1*β*. Once the 7′-OH was substituted by OCH_3_, the inhibitory activity would be reduced significantly. We could also conclude that 7 *R*, 8 *R*, 7′*R*, 9′*S* isomers (**1a**, **2a**) might have more advantages in inhibiting mRNA expression of pro-inflammatory cytokines than those of 7 *S*, 8 *S*, 7′*S*, 9′*R* configurations (**1b**, **2b**).

## Conclusion

In conclusion, for the first time we reported four novel cycloneolignans (**1a**, **1b**, **2a** and **2b**) with an unprecedented 9-O-9′−7, 9′-cyclo-8, 1′- neolignan skeleton containing a furan ring and a seven-member ring formed by two C-3 units. The unique skeleton enriches the chemical profile of natural cycloneolignan group. The key biosynthetic process was inferred as two steps of electrophilic additions from cinnamic aldehyde and a dehydration between 9-OH and 9′-OH. Bioassay results revealed that (±) verniciasin A might play a putative role in exerting anti-inflammatory effects exhibited by the herbal. Its significant inhibition effect against overactivated microglia could throw light on research about theraputic agents of AD. Moreover, the stereo-selectivity indicated 7 *R*, 8 *R*, 7′*R*, 9′*S* isomers were responsible for the main inhibiting effect, which might be caused by different steric interaction modes with iNOS.

## Methods

### Experimental procedures

ECD spectra were measured on JASCO pu-2080. Optical rotations were obtained by Perkin-Elmer Model 341 polarimeter. ^1^H-NMR and ^13^C-NMR spectra were collected on Bruker AV-400 NMR spectrometers and TMS was used as the internal standard. All the 2D NMR spectra were performed on Bruker AV-600 NMR spectrometers. HR-ESIMS data was afforded by Bruker micro-TOFQ-Q mass spectrometer. Regular column chromatography was carried out with Silica gel (48–75μm, Qingdao Marine Chemical Co. Ltd.). ODS (50 μm, YMC Co. Ltd., Kyoto, Japan) and Sephadex LH-20 (Amersham Pharmacia Biotech AB, Uppsala). HPLC was performed on a YMC ODS column (5 μm, 20 mm × 250 mm), using a Shimadzu LC-6AD pump and a RID-10A detector respectively. Chiral HPLC separation was conducted on a Daicel IF column (5 μm, 0.46 cm × 25 cm) with a LC-10AD pump system (Shimadzu) and a UV detector (SPD-20A). All the organic solvents (AR) were purchased from Yuwang Indusrial Co. Ltd (Shandong, China), and the solvents for HPLC were purchased from Sigma-Aldrich Co. LLC (USA). Dulbecco’s Modified Eagle’s Medium (DMEM) was produced by Gibco BRL (Grand Island, NY, USA). Fetal bovine serum (FBS) was purchased from Tianjinhaoyang Biological Manufactory Co., Ltd (Tianjin, China). Trizol was from Invitrogen Co. (Carlsbad, CA, USA). And (3-[4,5- dimethylthiazol-2-yl]−2,5- diphenyltetrazolium bromide (MTT) was purchased from Beyotime Biotechnology (Beijing, China). Lipopolysaccharide (LPS, from E. coli serotype 055:B5) was from Sigma Chemical Co. (St. Louis, MO, USA).

### Plant material

The seed capsules of *Vernicia fordii* were provided by Ningbolihua Plant Extraction Technology Co., Ltd. (Zhejiang, China). And the original plant was identified by Prof. Jincai Lu, School of Traditional Chinese Meteria Media, Shenyang Pharmaceutical University. The voucher specimen (20130913) was kept in School of Traditional Chinese Meteria Media, Shenyang Pharmaceutical University, Shenyang, China.

### Extraction and isolation

The air-dried seed capsules of *V. fordii* (3.2 kg) were powdered and extracted under reflux with 70% EtOH (3 × 2 h × 25 L). Then, the crude extract (320.0 g) was dissolved in 2 L hot water and participated with PE, EtOAc, *n*-BuOH respectively. And the EtOAc extract (222.0 g) was further isolated on silica gel column (110 × 10.5 cm), eluted by CH_2_Cl_2_/MeOH (100: 0–0: 100) to get Fr 2.1, 2.2, 2.3, 2.4 and 2.5. Then Fr 2.5 was further separated by ODS column chromatography eluted with MeOH-H_2_O (10: 90). And the elute was purified by HPLC with a ODS column and eluted by MeOH/H_2_O (18: 82) to obtain compound **1** (t_*R* = _29.0 min) and compound **2** (t_*R = *_44.1 min). Finally, chiral separation of **1** and **2** was achieved by HPLC with a Daicel IF column (*n*-hexane / EtOH / CF_3_COOH, 90: 10: 0.1). As a result, compound **1** was separated to afford compounds **1a** (t_*R* = _4 0.9 min) and **1b** (t_*R* = _47.4 min), while compound **2** was purified as compounds **2a** (t_*R* = _27.9 min) and **2b** (t_*R* = _31.4 min).

### Cell culture

The murine microglial cell line BV-2 was from Shanghai Institutes for Biological Sciences, Chinese Academy of Sciences (kindly provided by Prof. Y.Q. Guo, Nankai University, China). BV-2 cells were grown (37 °C and 5% CO_2_) in DMEM supplemented with 10% FBS, 100 U/ml penicillin and 100 μ g/ml streptomycin. Stock cells were passaged about three times/week and used within 8 passages.

### Determination of cell viability

Viability of BV2 cells was tested by MTT method described in our previous work^9–12^. The cells (2 × 10^4^ cells / well) were plated into 96-well plates and were pretreated with tested samples at different concentrations for 24 h. After the supernatant taken away, MTT (0.25 mg/ml) was added into the plate and incubated for 4 h at 37 °C. Finally, the formazan crystals were dissolved in DMSO and the absorbance of each well was recorded at 490 nm using a plate reader (Bio-Tek, Winooski, VT, USA).

### Nitrite assay

The production of NO in LPS-induced BV2 cells was assayed by Griess reaction^9–12^. The cells (2 × 10^4^ cells/well) were plated into 96-well plates and incubated with tested samples with 100 ng/mL LPS. Twenty-four hours later, Griess reagent was added into the supernatant at room temperature. And the plates were read on a plate reader at 540 nm after fifteen minutes.

### Measurement of IL-1β and IL-6 mRNA expression^9^

BV2 cells (4 × 10^5^ cells/ml) were plated into 6-well plates and were pretreated with each sample for 2 h and then induced by LPS for 4 h. After that the total RNA was isolated using Trizol and converted to cDNA. CFX ConnectTM real-time PCR detection system (Bio-Rad, Hercules, CA, USA) was used for the experiment. And the primers used are listed in Table 3. The level of GAPDH gene was used for standardization^9^.

**Table 3:** Primer sequence used in Real-time PCR assay.

Gene	Forward primer	Reverse primer
IL-1β	5′-TGACGGACCCCAAAAGATGA-3′	5′-TCTCCACAGCCACAATGAGT-3′
IL-6	5′-TAGTCCTTCCTACCCCAATTTCC-3′	5′-TTGGTCCTTAGCCACTCCTTC-3′
GAPDH	5′-AGGTCGGTGTGAACGGATTTG-3′	5′-TGTAGACCATGTAGTTGAGGTCA-3′

### Molecular docking

Molecular docking was performed according to the method described in our previous work.^9^ AutoDock4.2^38^ was used for the study. Firstly, the crystal structure of iNOS was extracted from the complex with a nanomolar imidazopyridine inhibitor (PDB code 3NW2)^39^. Then nine separate docking calculations were performed for each tested structure. Moreover, in order to check whether the docking model could reproduce the binding mode and binding affinity of known ligands, several active compounds collected from literature were docked onto iNOS pocket before docking our own compounds. After that, molecular docking was performed on the novel compounds. Discovery Studio Visualizer 2016 software package^40^ was used for molecular display.

### Statistical analysis

Statistical analyses were performed with SPSS 17.0 software (SPSS Inc., Chicago, IL, USA). The data were presented as mean ± SEM and analyzed using one-way analysis of variance (ANOVA). To compare groups, post hoc testing was carried out with Dunnett’s T3 test or Fisher’s least significant difference (LSD) test. Differences were considered statistically significant at P < 0.05.

## Electronic supplementary material

supporting information
