# Supplementary material for: Novel cycloneolignans from Vernicia fordii with inhibitory effects on over-activation of BV2 cells in vitro
Source: Sci Rep. 2017 Oct 19;7:13608. doi: 10.1038/s41598-017-14062-z (PMC5648763; doi:10.1038/s41598-017-14062-z)
Supplement: Supplementary file 1 — supporting information [file 41598_2017_14062_MOESM1_ESM.doc]

Supporting information

**Novel cycloneolignans from *Vernicia fordii* with inhibitory effects on over-activation of BV2 cells *in vitro***

Wei-Hong Zhao^1^, Ning Li^1,*^, Yang Chu^2^, Jian Wang^3^, Wen-Li Wang^1^, Jia-Yuan Li^1^, Bin Lin^3^, Ru Chen^4^, Yue Hou^4, *^

1 School of Traditional Chinese Materia Medica, Shenyang Pharmaceutical University; Key Laboratory of Structure-Based Drug Design and Discovery, Ministry of Education, 103 Wenhua Road, Shenyang 110016, China

2 Department of Pharmacy, the First Affiliated Hospital of China Medical University, 155 Nanjing Street, Shenyang

3 School of Pharmaceutical Engineering, Shenyang Pharmaceutical University; Key Laboratory of Structure-Based Drug Design and Discovery, Ministry of Education, 103 Wenhua Road, Shenyang 110016, China

4 College of Life and Health Sciences, Northeastern University, Shenyang, 110819, P.R. China

*corresponding.author: * [liningsypharm@163.com](mailto:liningsypharm@163.com)

* houyue@mail.neu.edu.cn

Contents

**E**xperimental Section.......................................................................................................................S2

Figure S1 The ^1^H-NMR spectrum of compd.1 measured in DMSO-*d*_6_..........................................S6

Figure S2 Expanded ^1^H-NMR spectrum of compd. 1 measured in DMSO-*d*_6_*.*...............................S7

Figure S3 The ^13^C-NMR spectrum of compd. 1 measured in DMSO-*d*_6_........................................S8

Figure S4 The HSQC spectrum of compd. 1 measured in DMSO-*d*_6_.............................................S9

Figure S5 The HMBC spectrum of compd. 1 measured in DMSO-*d*_6_..........................................S10

Figure S6 Expanded HMBC spectrum of compd. 1 measured in DMSO-*d*_6_................................S11

Figure S7 The NOESY spectrum of compd. 1 measured in DMSO-*d*_6_.........................................S12

Figure S8 Expanded NOESY spectrum of compd. 1 measured in DMSO-*d*_6_...............................S13

Figure S9 The HRESIMS spectrum of compd. 1 measured in CH_3_OH........................................S14

Figure S10 The ^1^H-NMR spectrum of compd. 2 measured in DMSO-*d*_6_.....................................S15

Figure S11 Expanded ^1^H-NMR spectrum of compd. 2 measured in DMSO-*d*_6_............................S16

Figure S12 The ^13^C-NMR spectrum of compd. 2 measured in DMSO-*d*_6_....................................S17

Figure S13 The HSQC spectrum of compd. 2 measured in DMSO-*d*_6_.........................................S18

Figure S14 The HMBC spectrum of compd. 2 measured in DMSO-*d*_6_........................................S19

Figure S15 Expanded HMBC spectrum of compd. 2 measured in DMSO-*d*_6_..............................S20

Figure S16 The NOESY spectrum of compd. 2 in measured DMSO-*d*_6_.......................................S21

Figure S17 Expanded NOESY spectrum of compd. 2 measured in DMSO-*d*_6_.............................S22

Figure S18 The HRESIMS spectrum of compd. 2 measured in CH_3_OH......................................S23

Figure S19 The chiral separation HPLC chromatogram of compd. 1 and 2..................................S24

Figure S20 Binding patterns of compounds from test set including 3 active compounds with diverse structure.................. ........................ ........................ ........................ ..............................S25

Figure S21 Docked poses for test set compounds were aligned within iNOS pocket..................S26

Figure S22 Docked poses for both test set compounds and our discovered natural products were aligned within iNOS pocket, to compare their binding patterns.......... ...... ..... ..... ..... ..... ..........S26

Fig S23 Alignment of docked pose (orange) against the experimental pose (green). Atoms were displayed as sticks. .......... ...... ..... ..... ..... ..... ......... .... ..... .... ..... .... ..... .... ..... .... ..... .... ........S27

Fig 24S Correlation between predicted binding energies and experimental activity data for known iNOS inhibitors .......... ...... ..... ..... ..... ..... ......... .... ..... .... ..... .... ..... .... ..... .... ..... .... ........S28

Table S1 Correlation between predicted binding energies and experimental activity data for known iNOS inhibitors .......... ...... ..... ..... ..... ..... ......... .... ..... .... ..... .... ..... .... ..... .... ..... .... ........S29

**Experimental Section**

**1. Chiral separation.** compounds **1** and **2** were dissolved in MeOH at 5mg/ml. Then, they were conducted on a Daicel IF column (5 μm, 0.46 cm×25 cm) with a LC-10AD pump system (Shimadzu) and a UV detector (SPD-20A). The column temperature was 25℃. Detection wavelength was 254 nm. Injection volume was 15μL. Later, compound **1** was separated into compounds **1a** (t_R_=40.9 min) and **1b** (t_R_=47.4 min) using *n*-hexane/EtOH/CF_3_COOH (90: 10: 0.1) as mobile phase, while compound **2** was purified as compounds **2a** (t_R_=27.9 min) and **2b** (t_R_=31.4 min) in the same chromatographic condition. In conclusion, four compounds, 7*R*, 8*R*, 7'*R*, 9'*S*-verniciasin A (**1a**), 7*S*, 8*S*, 7'*S*, 9'*R*- verniciasin A (**1b**), 7*R*, 8*R*, 7'*R*, 9'*S*-7'-methoxylverniciasin A (**2a**) and 7*S*, 8*S*, 7'*S*, 9'*R*-7'-methoxylverniciasin A (**2b**), were obtained from the husk of *Vernicia fordii*.

**2. ECD calculation methods**

Computational methods for ECD of compounds **1a** and **2a**

The 3D structures of the compounds were constructed with Discovery Studio 3.5 and were subjected to conformational search by Conflex 5c. Due to the existence of the ether bridge, the compounds were relatively constrained therefore only one dorminant conformation was located for each compound. They were used as the input for the structural optimization by the density functional theory (DFT) method at the B3LYP/6-31G(d) level in Gaussian 09 program package.^[1]^ The optimized conformations were checked by frequency analysis and no imaginary frequencies were found. The ECD of the conformers of 1a and 1b were then calculated by the TDDFT method at the B3LYP/6-311++G (2d,p) level with the CPCM model in methanol solution. The calculated ECD curve was generated using SpecDis 1.6.2.^[2]^.

The energy is -1223.44792623 Hartrees for compound **1a** and -1262.74907204 Hartrees for compound **1b**.

Standard orientation for **1a:**

Standard orientation:

---------------------------------------------------------------------

Center Atomic Atomic Coordinates (Angstroms)

Number Number Type X Y Z

---------------------------------------------------------------------

1 1 0 0.252811 0.060298 -1.121288

2 6 0 0.627178 -0.407336 -0.207743

3 6 0 2.093530 -0.032880 -0.061152

4 6 0 2.628263 0.935623 -0.927720

5 6 0 3.954468 1.341393 -0.825353

6 8 0 4.444907 2.288276 -1.678079

7 6 0 4.786897 0.771111 0.155499

8 8 0 6.075774 1.241185 0.167411

9 6 0 4.277314 -0.195508 1.013958

10 6 0 2.938833 -0.594529 0.909974

11 6 0 0.360208 -1.936092 -0.261845

12 8 0 1.359026 -2.685606 -0.888634

13 6 0 -0.944532 -2.305344 -0.982551

14 6 0 -2.255211 -1.991850 -0.234713

15 1 0 -2.192853 -2.470409 0.753011

16 8 0 -3.342014 -2.581391 -0.957584

17 6 0 -2.573458 -0.511680 -0.035550

18 6 0 -1.671783 0.384553 0.580443

19 6 0 -2.068330 1.715843 0.779187

20 6 0 -3.319962 2.176360 0.386252

21 8 0 -3.670149 3.481709 0.596856

22 6 0 -4.208650 1.284213 -0.227951

23 8 0 -5.423575 1.817139 -0.588411

24 6 0 -3.835609 -0.039565 -0.427017

25 6 0 -0.259226 0.011065 1.002199

26 1 0 0.178984 0.879822 1.498992

27 6 0 -0.145591 -1.224453 1.933280

28 8 0 0.284833 -2.331865 1.128218

29 1 0 2.010135 1.391221 -1.696295

30 1 0 5.381328 2.431307 -1.452380

31 1 0 6.587377 0.800645 0.864669

32 1 0 4.921712 -0.639236 1.769106

33 1 0 2.566318 -1.352421 1.590657

34 1 0 2.218001 -2.352536 -0.570675

35 1 0 -0.967728 -1.842002 -1.975001

36 1 0 -0.911734 -3.392543 -1.128130

37 1 0 -3.211175 -3.542138 -0.932632

38 1 0 -1.388733 2.421532 1.249035

39 1 0 -4.573339 3.600695 0.253458

40 1 0 -5.975264 1.134047 -1.001837

41 1 0 -4.534557 -0.727085 -0.890187

42 1 0 0.601156 -1.059328 2.718274

43 1 0 -1.098893 -1.462973 2.417838

---------------------------------------------------------------------

The coordinates for the optimized conformation of compound **2a**:

Standard orientation:

---------------------------------------------------------------------

Center Atomic Atomic Coordinates (Angstroms)

Number Number Type X Y Z

---------------------------------------------------------------------

1 1 0 0.420690 0.175790 -1.107070

2 6 0 0.759610 -0.294990 -0.181410

3 6 0 2.252990 -0.041190 -0.051290

4 6 0 2.863770 0.847970 -0.951910

5 6 0 4.221750 1.136900 -0.871000

6 8 0 4.786600 2.007959 -1.757850

7 6 0 5.009460 0.525709 0.122050

8 8 0 6.335450 0.877689 0.108760

9 6 0 4.423340 -0.362650 1.015600

10 6 0 3.053830 -0.643950 0.932760

11 6 0 0.365070 -1.797340 -0.191670

12 8 0 1.296270 -2.645270 -0.796720

13 6 0 -0.968350 -2.075200 -0.901370

14 6 0 -2.243130 -1.642270 -0.150420

15 1 0 -2.209820 -2.096410 0.850160

16 8 0 -3.371520 -2.180250 -0.845320

17 6 0 -3.745220 -3.483800 -0.428790

18 6 0 -2.440970 -0.133060 0.006590

19 6 0 -1.463970 0.704430 0.590180

20 6 0 -1.750660 2.068390 0.753940

21 6 0 -2.964050 2.617650 0.355220

22 8 0 -3.206530 3.952290 0.531260

23 6 0 -3.926510 1.783310 -0.228360

24 8 0 -5.097420 2.401910 -0.597180

25 6 0 -3.663310 0.428650 -0.391260

26 6 0 -0.083230 0.230290 1.017380

27 1 0 0.425810 1.075060 1.487440

28 6 0 -0.066400 -0.982790 1.984290

29 8 0 0.260530 -2.145780 1.209540

30 1 0 2.282210 1.330570 -1.732460

31 1 0 5.734860 2.071269 -1.545380

32 1 0 6.817250 0.400939 0.803570

33 1 0 5.032240 -0.837651 1.781040

34 1 0 2.622310 -1.344510 1.639430

35 1 0 2.180250 -2.378430 -0.484740

36 1 0 -0.960290 -1.624550 -1.899760

37 1 0 -1.022350 -3.161720 -1.031440

38 1 0 -4.016370 -3.502830 0.636920

39 1 0 -4.617090 -3.766380 -1.024390

40 1 0 -2.948760 -4.221560 -0.600450

41 1 0 -1.013450 2.729260 1.201670

42 1 0 -4.100420 4.133730 0.190870

43 1 0 -5.708370 1.751610 -0.978980

44 1 0 -4.417490 -0.214880 -0.830620

45 1 0 0.699430 -0.859000 2.758590

46 1 0 -1.031810 -1.125040 2.482560

---------------------------------------------------------------------


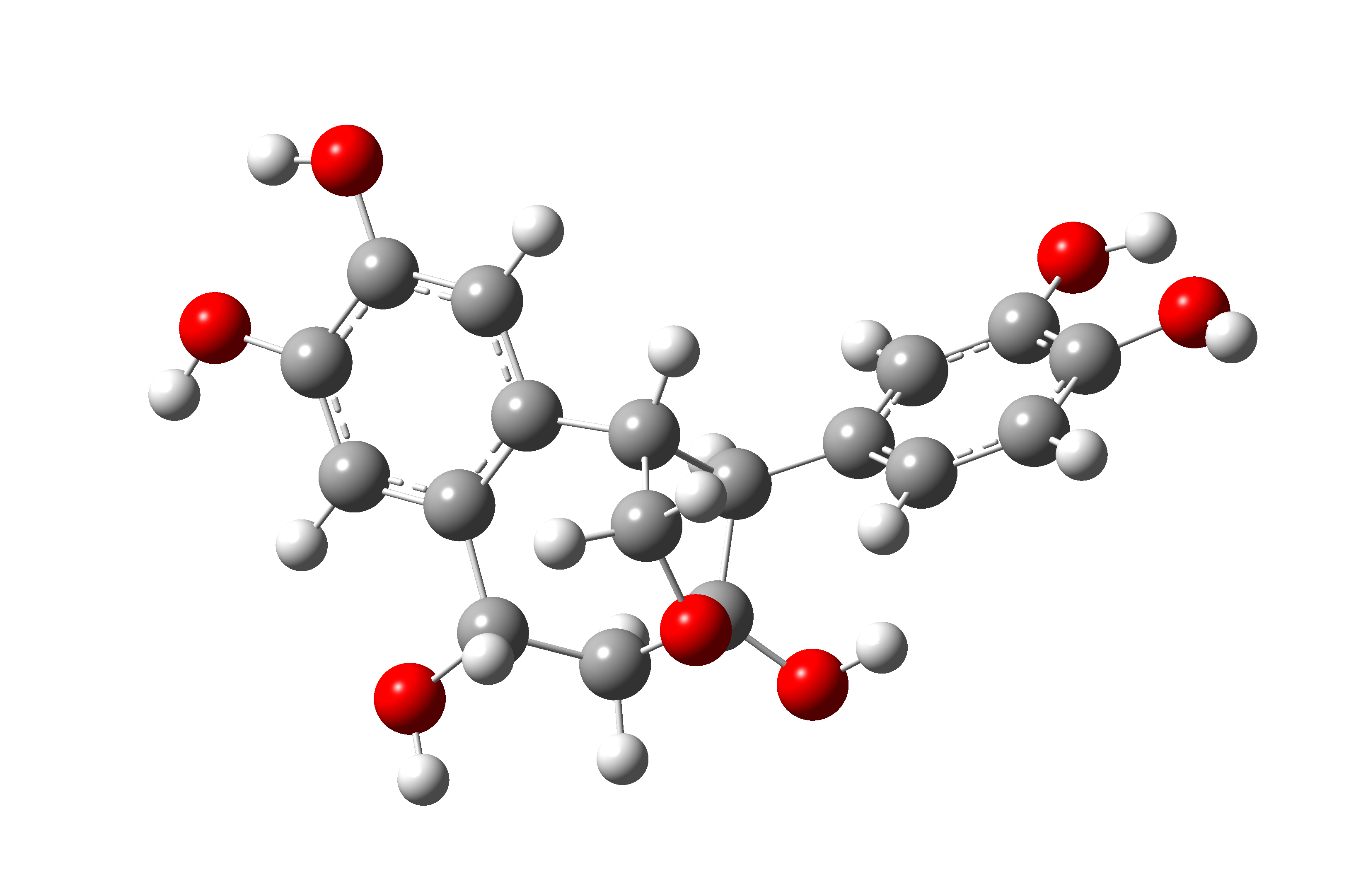

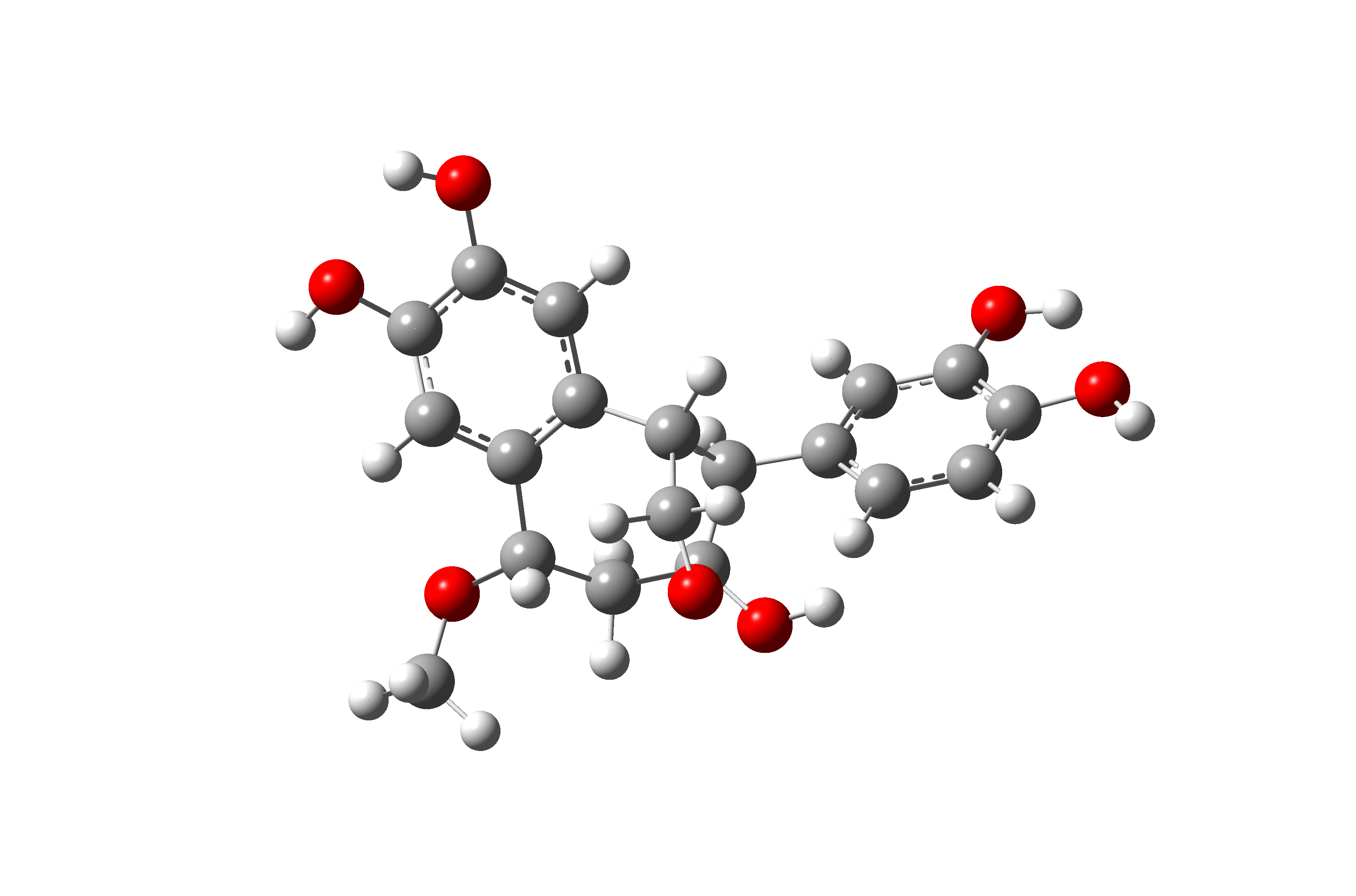


7*R*, 8*R*, 7'*R*, 9'*S*-verniciasin A (**1a**) 7*R*, 8*R*, 7'*R*, 9'*S*-7′-methoxylverniciasin A (**2a**)

B3LYP/6-31+G** optimized lowest energy 3D conformer of 7*R*, 8*R*, 7'*R*, 9'*S*-verniciasin A (**1a**) and 7*R*, 8*R*, 7'*R*, 9'*S*-7′-methoxylverniciasin A (**2a**)

**References**

[1] Frisch, M. J.; Trucks, G. W.; Schlegel, H. B.; Scuseria, G. E.; Robb, M. A.; Cheeseman, J. R.; Scalmani, G.; Barone, V.; Mennucci, B.; Petersson, G. A.; Nakatsuji, H.; Caricato, M.; Li, X.; Hratchian, H. P.; Izmaylov, A. F.; Bloino, J.; Zheng, G.; Sonnenberg, J. L.; Hada, M.; Ehara, M.; Toyota, K.; Fukuda, R.; Hasegawa, J.; Ishida, M.; Nakajima, T.; Honda, Y.; Kitao, O.; Nakai, H.; Vreven, T.; Montgomery, J. A., Jr.; Peralta, J. E.; Ogliaro, F.; Bearpark, M.; Heyd, J. J.; Brothers, E.; Kudin, K. N.; Staroverov, V. N.; Kobayashi, R.; Normand, J.; Raghavachari, K.; Rendell, A.; Burant, J. C.; Iyengar, S. S.; Tomasi, J.; Cossi, M.; Rega, N.; Millam, J. M.; Klene, M.; Knox, J. E.; Cross, J. B.; Bakken, V.; Adamo, C.; Jaramillo, J.; Gomperts, R.; Stratmann, R. E.; Yazyev, O.; Austin, A. J.; Cammi, R.; Pomelli, C.; Ochterski, J. W.; Martin, R. L.; Morokuma , K . ; Zakrzewski, V. G .; Voth, G. A.; Salvador , P .; Dannenberg,J.J.; Dapprich,S.; Daniels,A.D.; Farkas,O; Foresman, J. B.; Ortiz, J. V.; Cioslowski, J.; Fox, D. J. Gaussian 09, Revision C1; Gaussian, Inc.: Wallingford, CT, **2010**.

[2] Bruhn, T.; Schaumlöffel, A.; Hemberger, Y.; Bringmann, G. *Chirality* **2013**, *25*, 243-249.


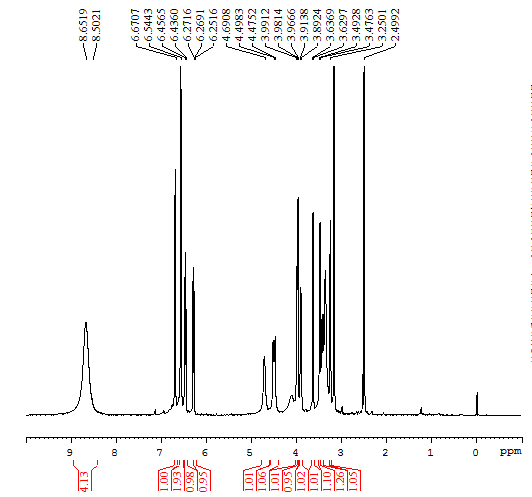


**Figure S1.** The ^1^H-NMR spectrum of compd. **1** measured in DMSO-*d*_6_ (400 MHz)


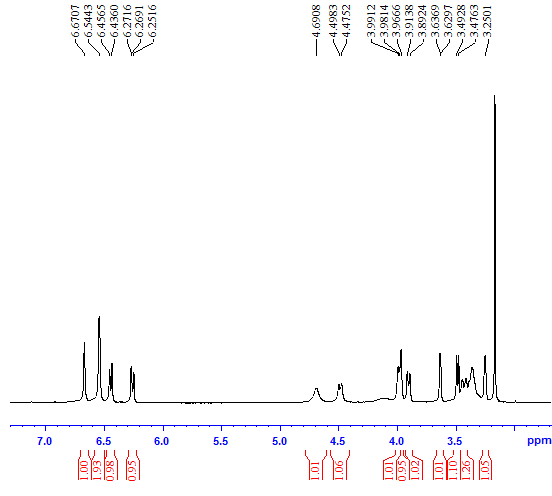


**Figure S2.** Expanded ^1^H-NMR spectrum of compd.**1** measured in DMSO-*d*_6_ (400 MHz)


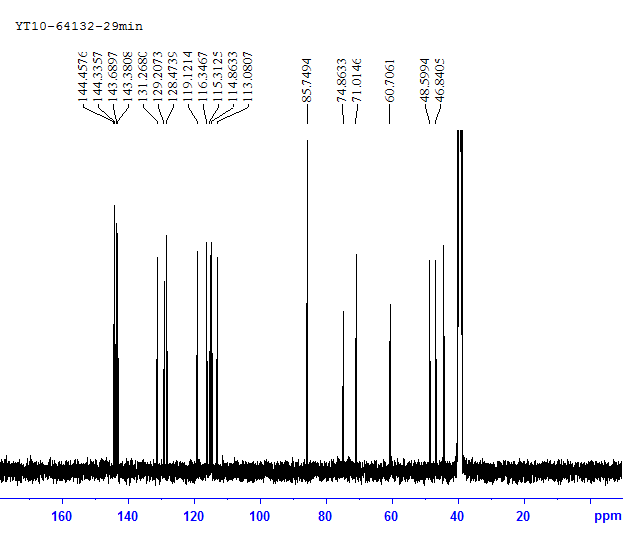


**Figure S3.** The ^13^C-NMR spectrum of compd. **1** measured in DMSO-*d*_6_ (100 MHz)


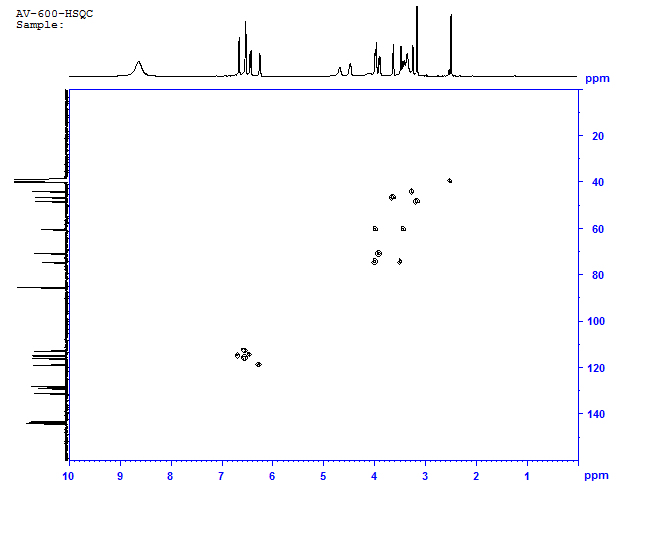


**Figure S4.** The HSQC spectrum of compd. **1** measured in DMSO-*d*_6_ (600 MHz)


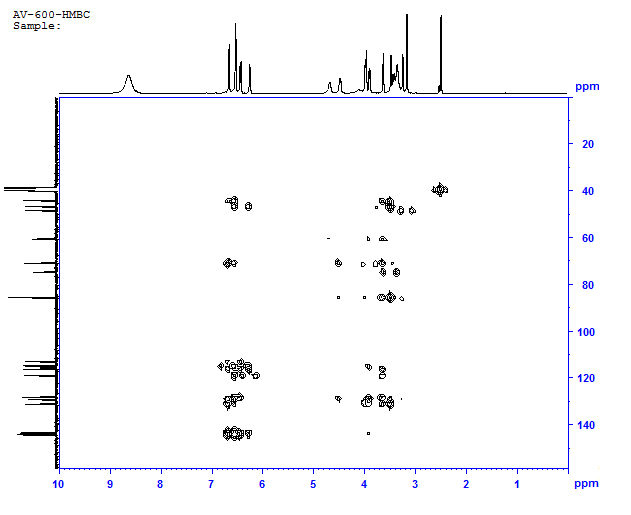


**Figure S5.** The HMBC spectrum of compd. **1** measured in DMSO-*d*_6_ (600 MHz)


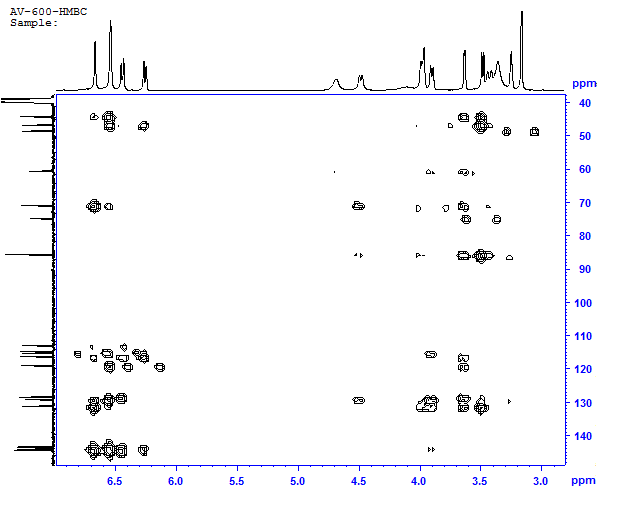


**Figure S6.** Expanded HMBC spectrum of compd. **1** measured in DMSO-*d*_6_ (600 MHz)

**
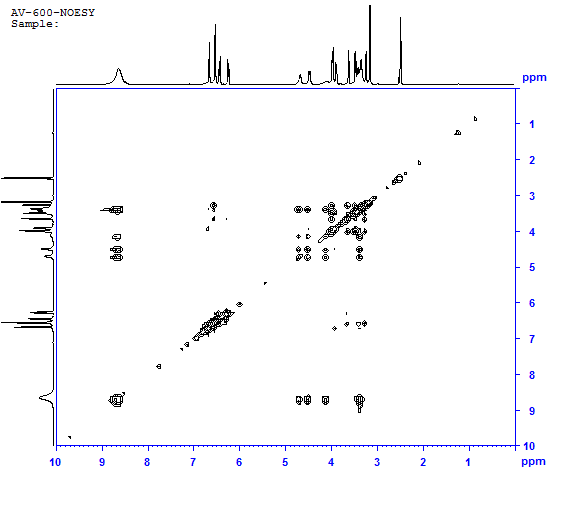
**

**Figure S7.** The NOESY spectrum of compd. **1** measured in DMSO-*d*_6_ (600 MHz)


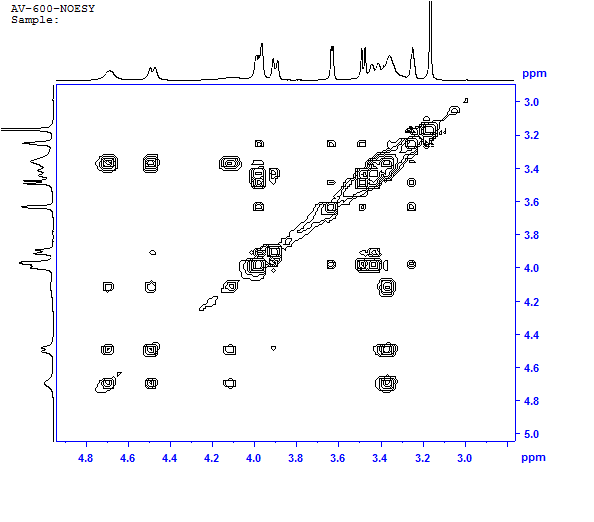


**Figure S8.** Expanded NOESY spectrum of compd. **1** measured in DMSO-*d*_6_ (600 MHz)


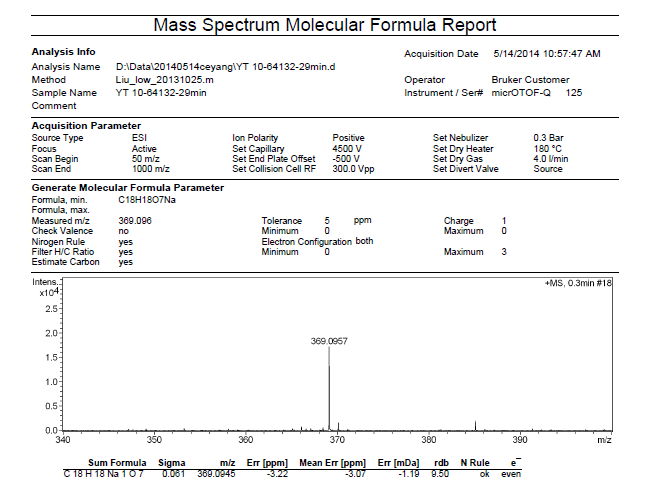


**Figure S9.** The HRESIMS spectrum of compd. **1** measured in CH_3_OH


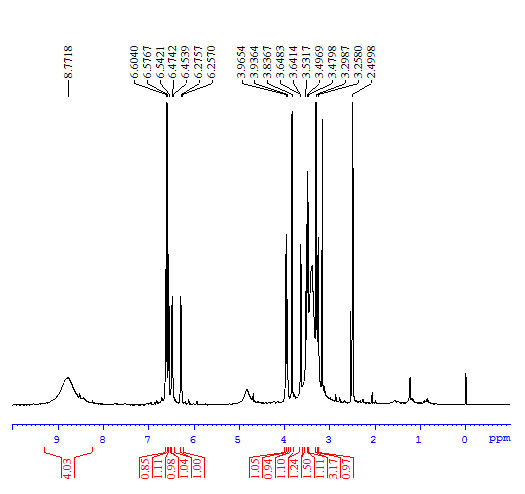


**Figure S10.** The ^1^H-NMR spectrum of compd. **2** measured in DMSO-*d*_6_ (400 MHz)


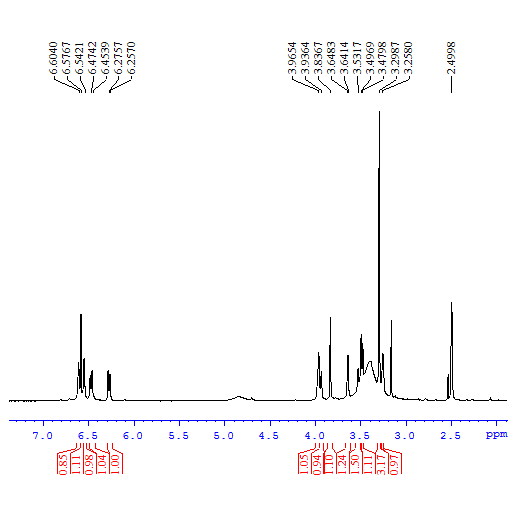


**Figure S11.** Expanded ^1^H-NMR spectrum of compd. **2** measured in DMSO-*d*_6_ (400 MHz)


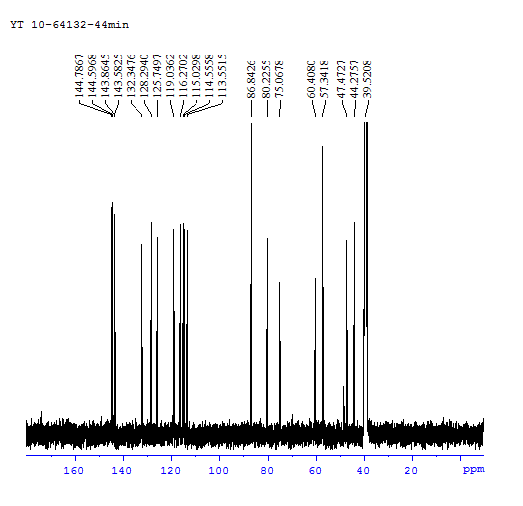


**Figure S12.** The ^13^C-NMR spectrum of compd. **2** measured in DMSO-*d*_6_ (100 MHz)


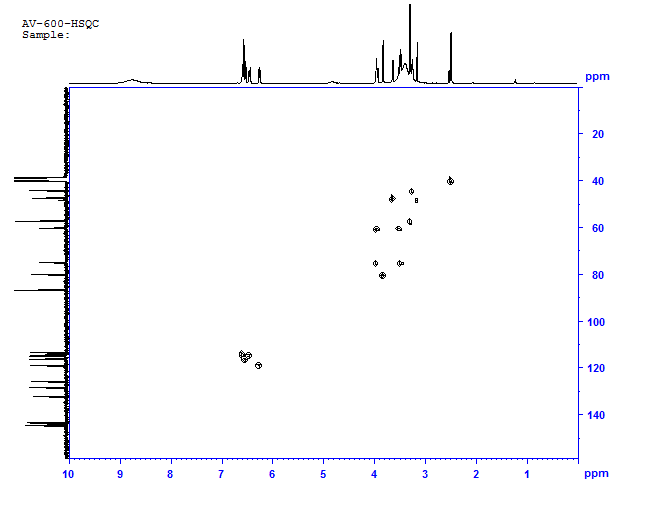


**Figure S13.** The HSQC spectrum of compd. **2** measured in DMSO-*d*_6_ (600 MHz)


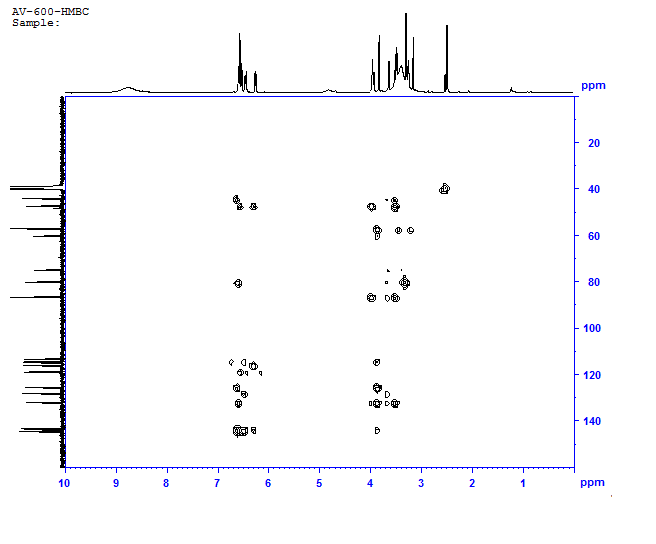


**Figure S14.** The HMBC spectrum of compd. **2** measured in DMSO-*d*_6_ (600 MHz)


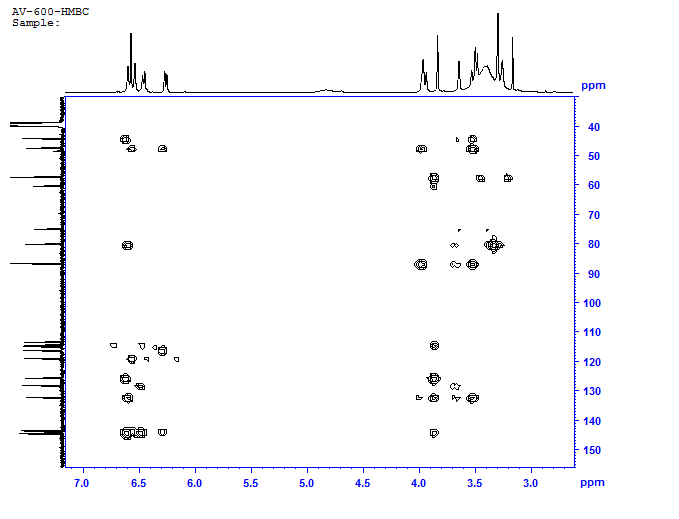


**Figure S15.** Expanded HMBC spectrum of compd. **2** measured in DMSO-*d*_6_ (600 MHz)


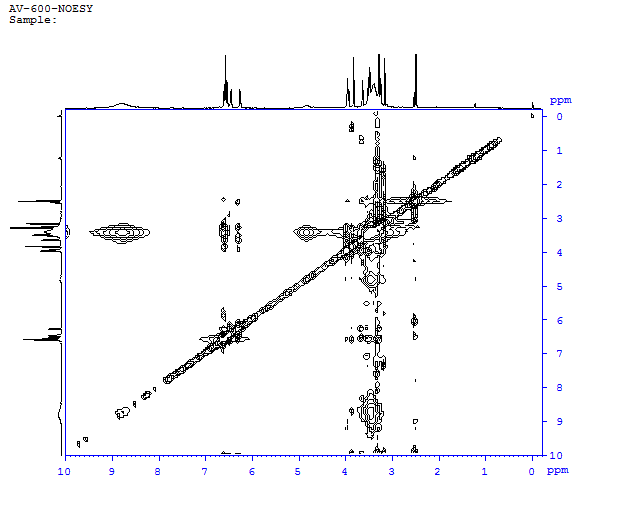


**Figure S16.** The NOESY spectrum of compd. **2** measured in DMSO-*d*_6_ (600 MHz)


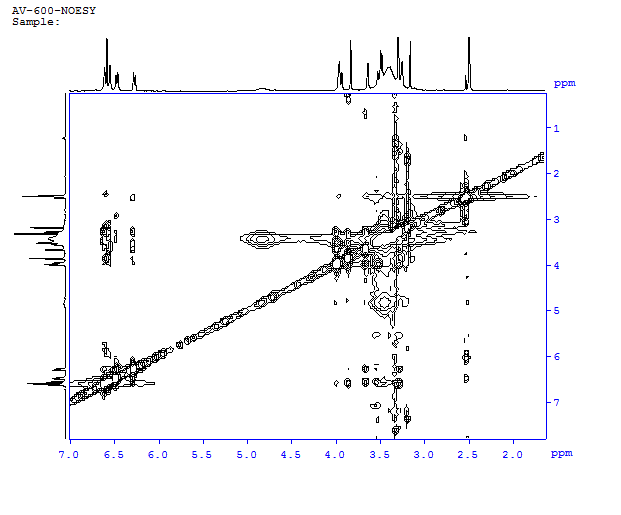


**Figure S17.** Expanded NOESY spectrum of compd. **2** measured in DMSO-*d*_6_ (600 MHz)


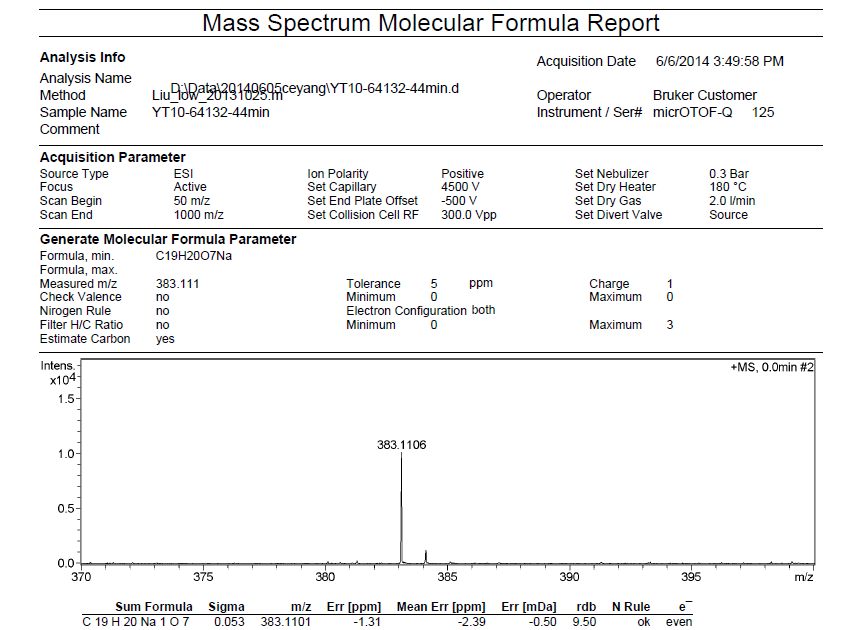


**Figure S18.** The HRESIMS spectrum of compd. **2** measured in CH_3_OH

**2a 2b 1a 1b**


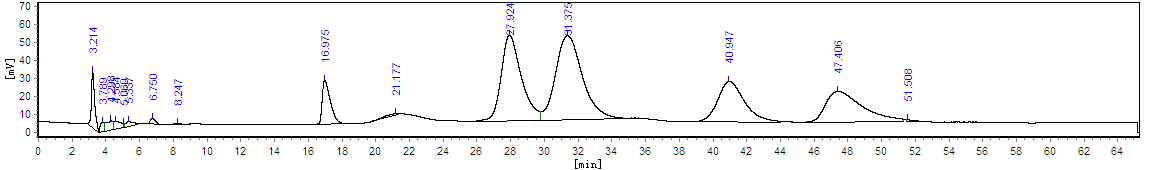


**Figure S19.** The chiral separation HPLC chromatogram of compounds **1** (**1a**, **1b**）and **2** (**2a**, **2b**)


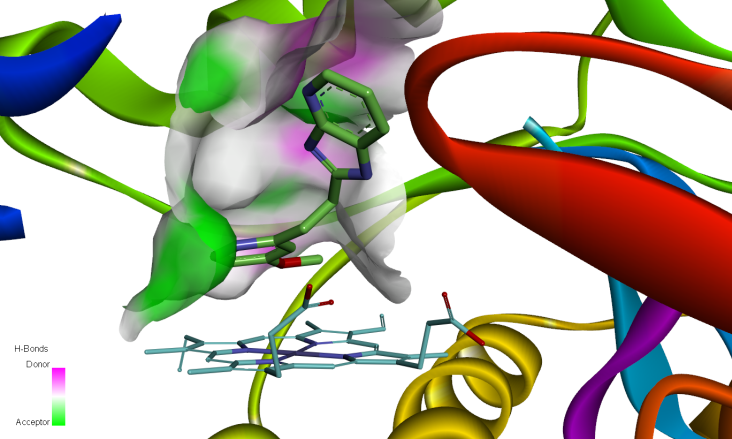


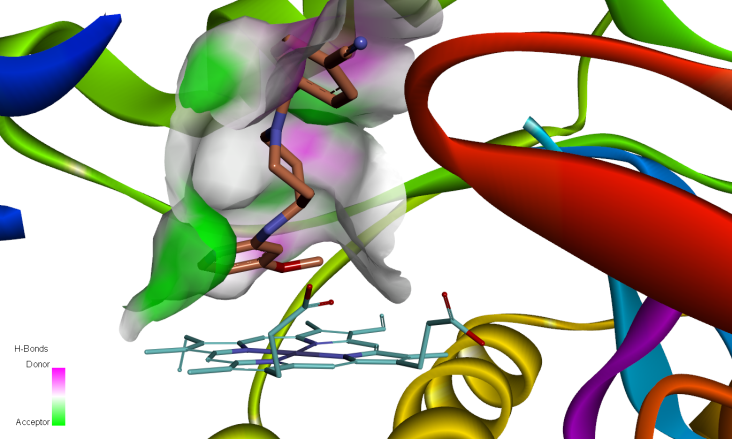


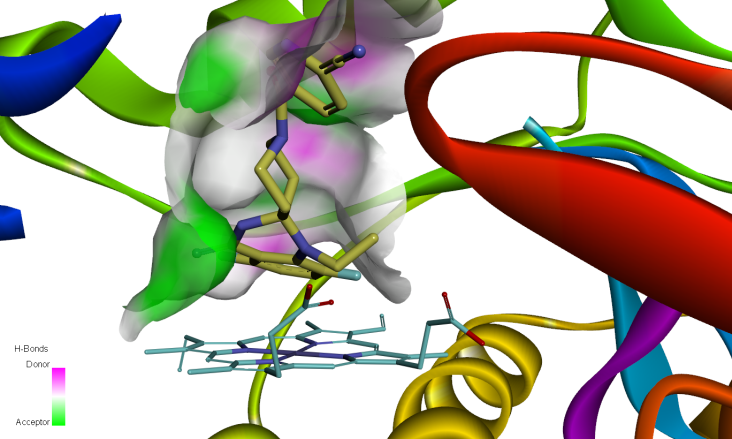


**Figure S 20** Binding patterns of compounds from test set including 3 active compounds with diverse structure. (The protein structure was shown as ribbon, compounds were displayed as sticks, HEME and residues were displayed as thin stick.)


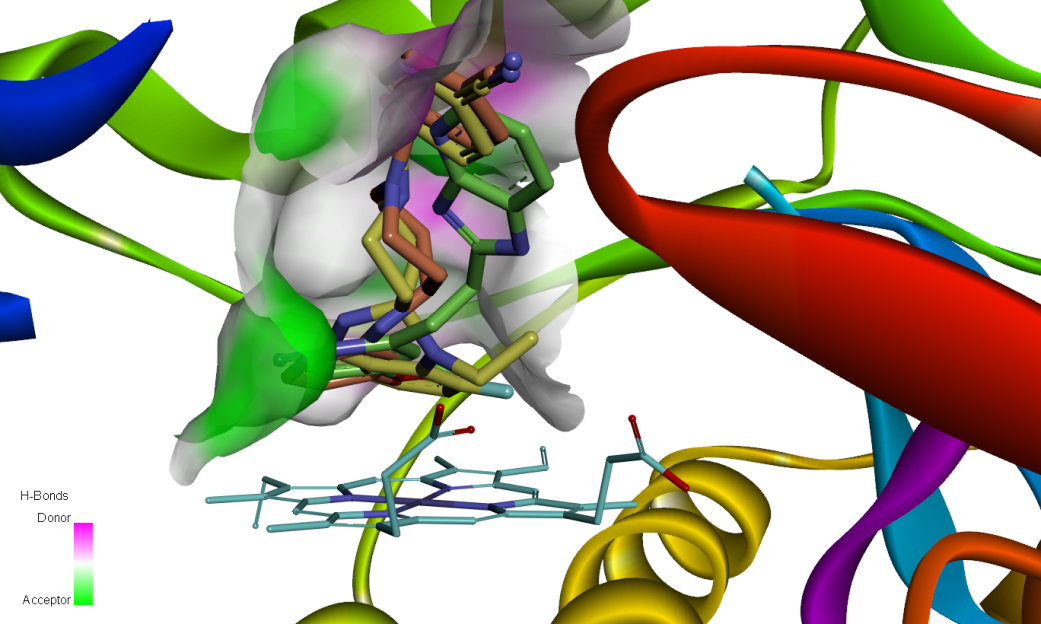

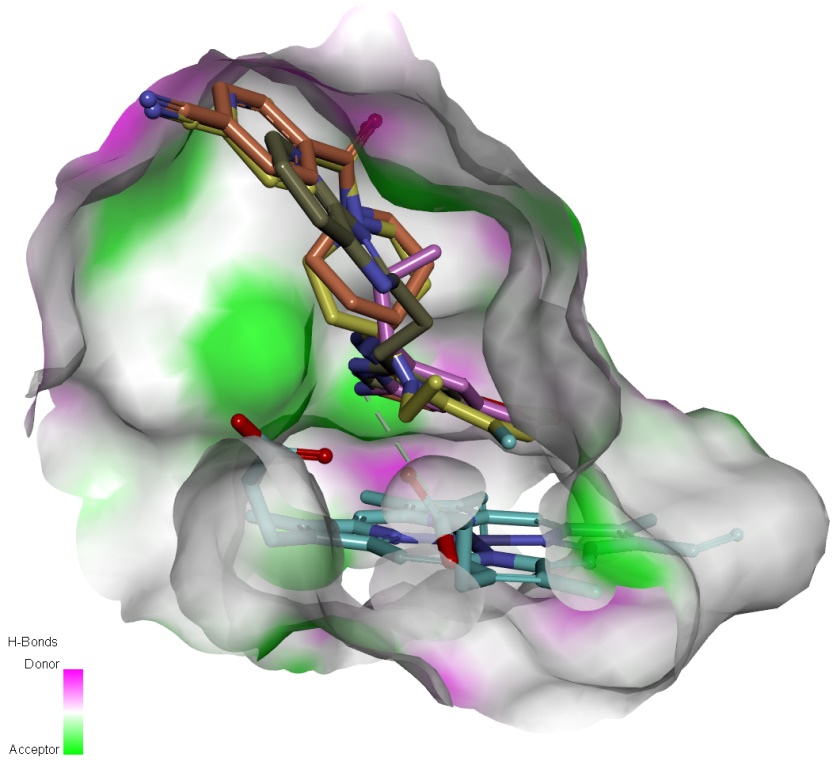


**Figure S 21** Docked poses for test set compounds were aligned within iNOS pocket


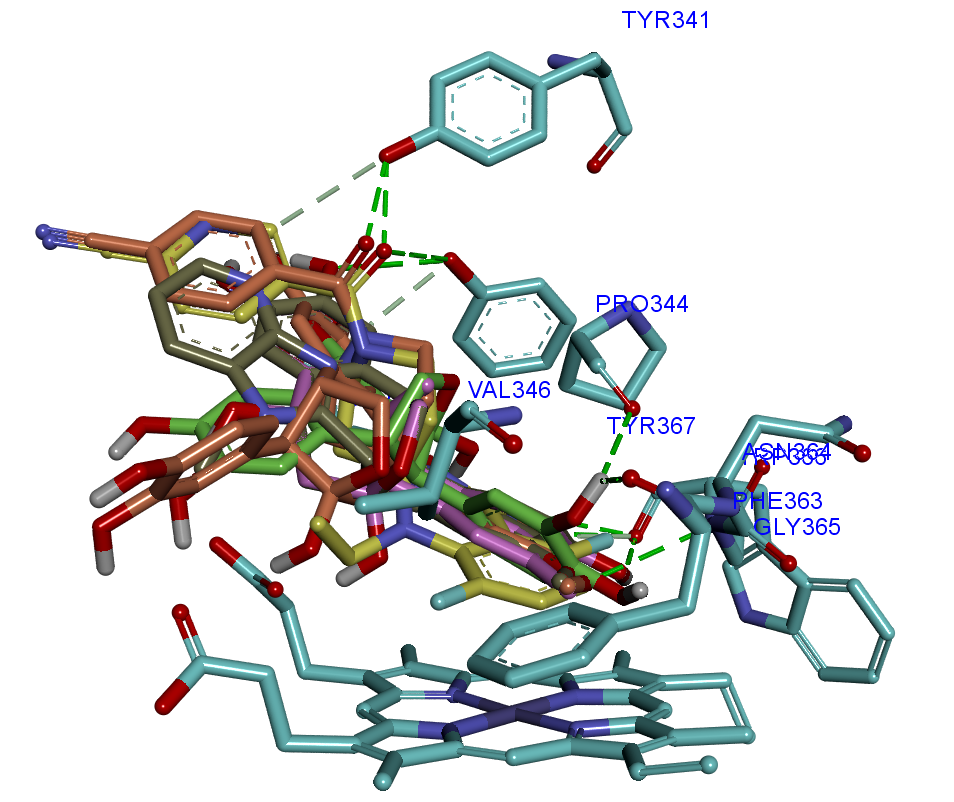

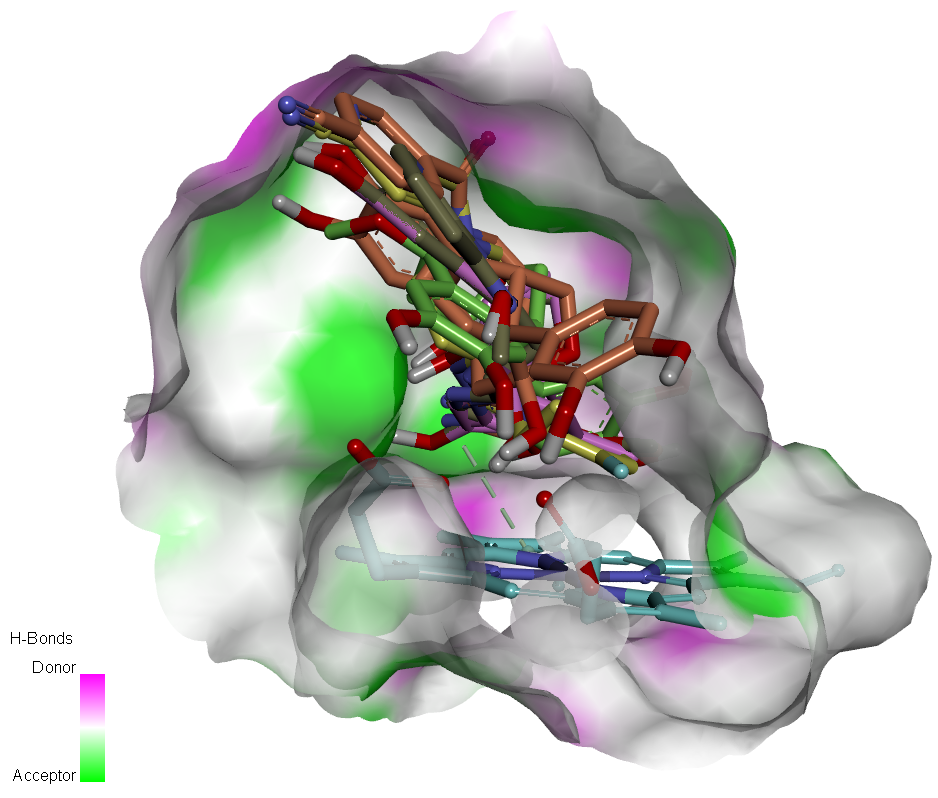


**Figure S 22** Docked poses for both test set compounds and our discovered natural products were aligned within iNOS pocket, to compare their binding patterns


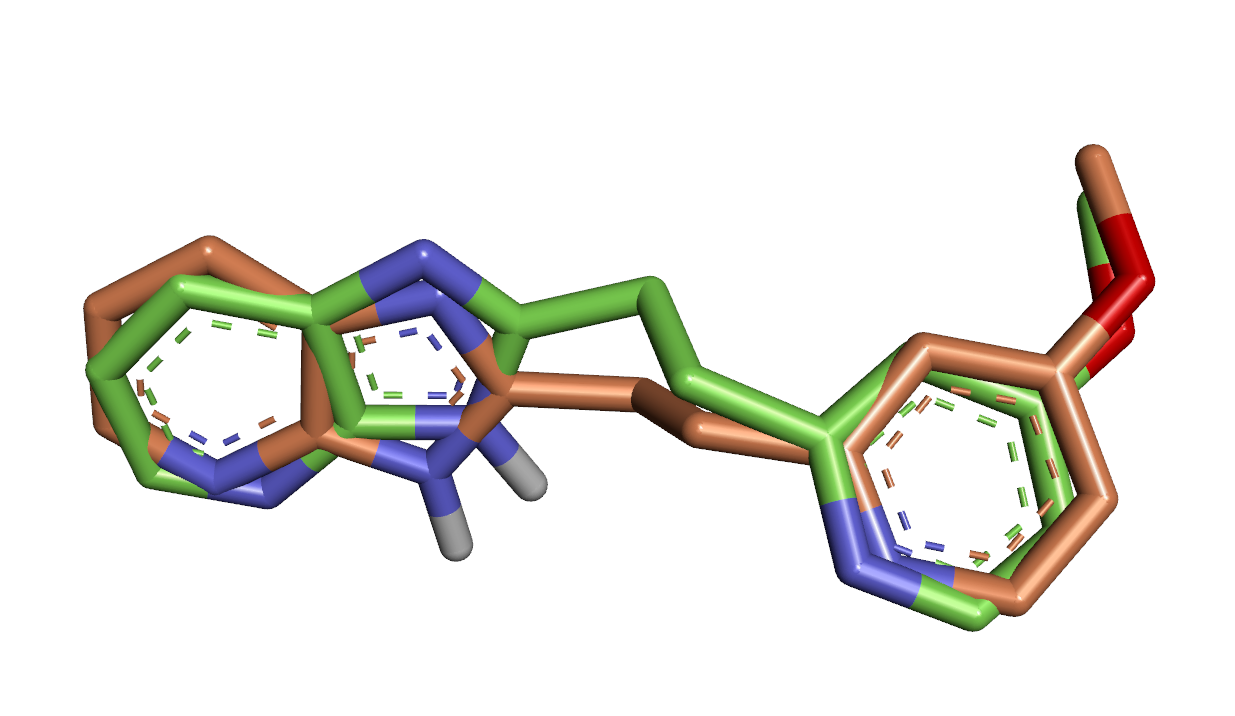


**Fig S23** Alignment of docked pose (orange) against the experimental pose (green). Atoms were displayed as sticks. (Garcin ,E.D.et al.Anchored plasticity opens doors for selective inhibitor design in nitric oxide synthase.Nat Chem Biol. 2008, 4,700-7)


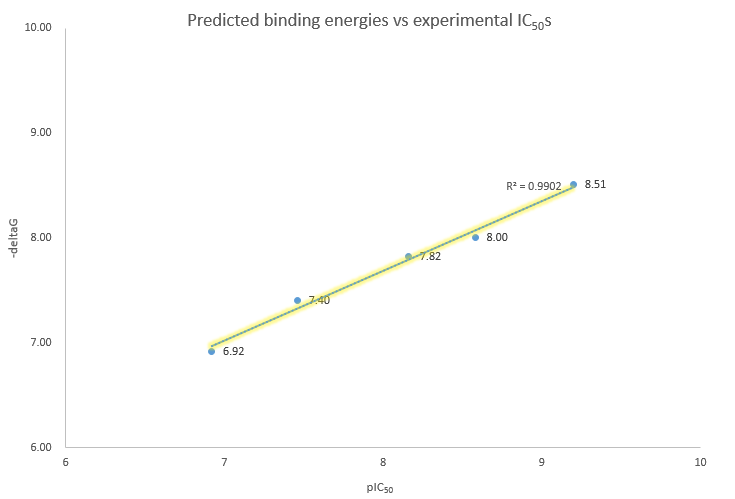


**Fig 24S** Correlation between predicted binding energies and experimental activity data for known iNOS inhibitors (Garcin ,E.D.et al.Anchored plasticity opens doors for selective inhibitor design in nitric oxide synthase.Nat Chem Biol. 2008, 4,700-7)

**Table S1** Correlation between predicted binding energies and experimental activity data for known iNOS inhibitors (Garcin ,E.D.et al.Anchored plasticity opens doors for selective inhibitor design in nitric oxide synthase.Nat Chem Biol. 2008, 4,700-7)

| structure | IC_50_ (nM) | _p_IC_50_ | Binding Energy (△G, kcal/mol) | -△G(kcal/mol) |
| --- | --- | --- | --- | --- |
|  | 10 | 8.00 | -8.58 | 8.58 |
|  | 31 | 8.51 | -9.20 | 9.20 |
|  | 120 | 6.92 | -6.92 | 6.92 |
|  | 15 | 7.82 | -8.16 | 8.16 |
|  | 40 | 7.40 | -7.46 | 7.46 |
